# Size Selective Ligand Tug of War Strategy to Separate Rare Earth Elements

**DOI:** 10.1021/jacsau.2c00671

**Published:** 2023-01-25

**Authors:** Katherine R. Johnson, Darren M. Driscoll, Joshua T. Damron, Alexander S. Ivanov, Santa Jansone-Popova

**Affiliations:** † Nuclear Energy and Fuel Cycle Division, Oak Ridge National Laboratory, Oak Ridge, Tennessee 37831, United States; ‡ Chemical Sciences Division, Oak Ridge National Laboratory, Oak Ridge, Tennessee 37831, United States

**Keywords:** lanthanide, rare earth element, separation, BLPhen, diglycolamide

## Abstract

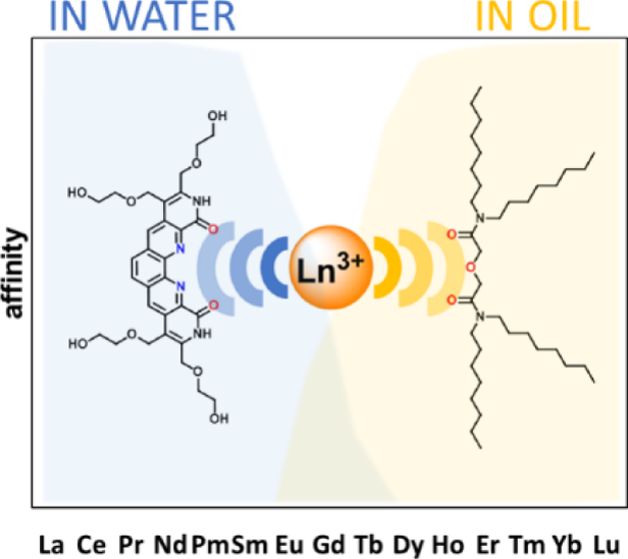

Separating rare earth elements is a daunting task due to their similar properties. We report a “tug of war” strategy that employs a lipophilic and hydrophilic ligand with contrasting selectivity, resulting in a magnified separation of target rare earth elements. Specifically, a novel water-soluble bis-lactam-1,10-phenanthroline with an affinity for light lanthanides is coupled with oil-soluble diglycolamide that selectively binds heavy lanthanides. This two-ligand strategy yields a quantitative separation of the lightest (e.g., La–Nd) and heaviest (e.g., Ho–Lu) lanthanides, enabling efficient separation of neighboring lanthanides in-between (e.g., Sm–Dy).

## Introduction

Yttrium, scandium, and the 15 lanthanides, known collectively as the rare earth elements (REEs), possess unique properties that make them indispensable materials in numerous applications and modern technologies.^1−6^ Additionally, the synthetic short-lived radioactive isotopes of REEs, such as Tb-161 and Lu-177, are used in radiopharmaceutical therapy to treat cancer.^7−9^ As such, the demand for REEs is increasingly affecting the supply chain, prompting improved strategies to recover and separate them from conventional and unconventional sources.^10−14^ Similarly, the isolation of target radioisotopes after neutron irradiation of adjacent stable isotopes (e.g., Tb-161 from Gd) requires the development of more efficient separations processes.^15−18^ The efficient separation of adjacent lanthanides remains a formidable challenge, and with growing demand for critical materials (i.e., Nd, Pr, and Dy) used in clean energy technologies, advancements in this field are desperately needed.^19−24^ The implemented processes on an industrial scale lack the selectivity needed to achieve efficient, environmentally sound, and cost-effective separation of lanthanides.^13,25^

The tandem use of hydrophilic and lipophilic ligands to separate REEs has been rarely investigated,^26−31^ despite being a common strategy employed to study separation of 4f and 5f elements.^32−43^ Such hydrophilic ligands, also known as holdback agents or aqueous complexants, include polyaminocarboxylates,^28−30^ α-hydroxy acids,^31^ diglycolamides (DGAs),^26,27^ and pyridine-based substrates,^37−41^ among others. In general, ligands with donor groups connected to freely rotating single bonds that can achieve high complementarity with metal ions (e.g., DGAs) exhibit high affinities for lanthanides that are more Lewis acidic.^42−49^ A combination of lipophilic DGA with hydrophilic dioxaoctanediamide results in improved selectivity for Gd over La (SF_Gd/La_ = 100 or 1130 based on the size of N-alkyl substituents on DGAs).^26,27^ This improvement is driven by dioxaoctanediamide exhibiting opposite selectivity to that of DGA, but is limited due to dioxaoctanediamide being a weaker extractant than DGA.^50^ Other ligands that show opposite selectivity across the trivalent lanthanide (Ln) series include substrates that incorporate conformational rigidity.^48,51−59^ For example, lipophilic bis-lactam-1,10-phenanthrolines (BLPhen)^51,52^ and macrocycles (macropa,^53,54^ macrophosphi,^57^ and py-macrodipa^58^) show high affinity for trivalent lanthanides having larger ionic radius. The latter are efficient chelators for radioisotopes in nuclear medicine applications at physiological pH.^58,60,61^ Using neutral extractants allows for operating at a more comprehensive pH range. The extraction of REEs is sensitive to changes in anion concentration when using neutral ligands, as the anions accompany Ln ions in the partition process. In contrast, variations in pH affect the cation exchange when utilizing organic acids.

**Figure 1:**
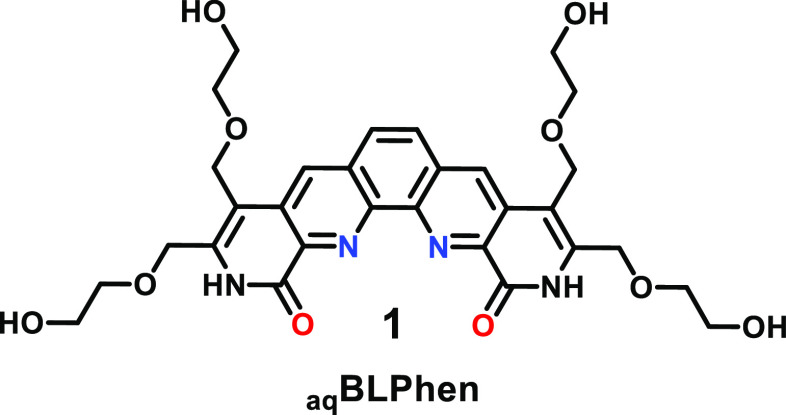
Chemical structure of _aq_BLPhen **1**.

We hypothesized that the addition of hydrophilic substituents on BLPhen will render this neutral ligand soluble in water (**1**, Figure 1) while maintaining the same general size selectivity across the Ln series^51^ and in combination with oil-soluble DGA would yield a synergistic process that separates Lns with improved selectivity. Herein, we present the development and performance of a synergistic separation system that employs _aq_BLPhen and DGA with contrasting selectivity and investigation of _aq_BLPhen-Ln complexes in aqueous solutions using spectroscopic methods.

## Results and Discussion

### Synthesis

**Figure 2:**
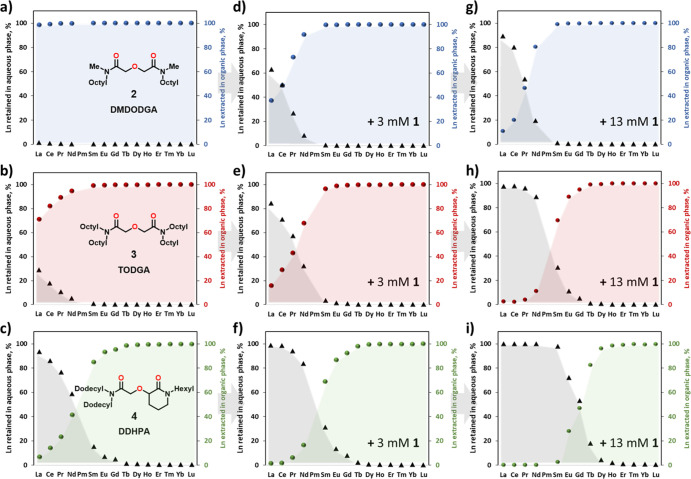
Chemical structures of DGAs **2**, **3**, and **4**. Graphs present the calculated variation in the extraction of 14 Lns (excluding *Pm*), 0.5 mM each, with 0.1 M **2**, **3**, and **4** from 1 M HNO_3_ (pH 0) into *n*-dodecane with 10 vol % 1-octanol at 25.5 ± 0.5 °C after 1 h in the absence (a–c) and presence of **1**, 3 mM (d–f) and 13 mM (g–i).

**Figure 3:**
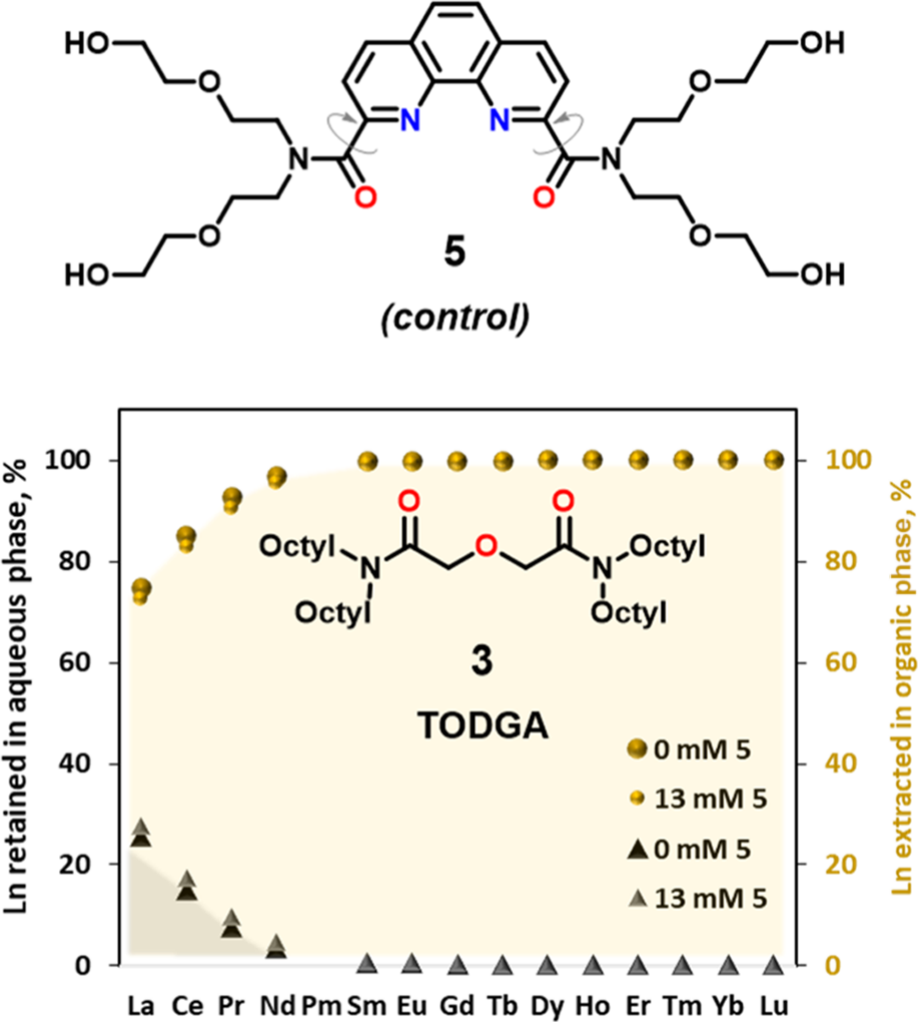
Chemical structure of hydrophilic ligand **5**. Variation in the extraction of 14 Lns (excluding *Pm*), 0.5 mM each, with 0.1 M **3** from 1 M HNO_3_ into *n*-dodecane with 10 vol % 1-octanol at 25.5 ± 0.5 °C after 1 h in the absence (0 mM) and presence of 13 mM **5**.

Water solubilizing ethylene glycol units were introduced in **1** via rhodium catalyzed C–H annulation reaction using 1,4-bis(2-hydroxyethoxy)-2-butyne as a reagent. The incorporation of ethylene glycol is a commonly employed strategy to improve the substrate’s solubility in aqueous media.^62,63^ The solubility of **1** in 0.9 M nitric acid (HNO_3_) is >0.2 M. Three DGAs were selected for this study (Figure 2): *N*,*N*′-dimethyl-*N*,*N*′-di(*n*-octyl)diglycolamide (DMDODGA, **2**), *N*,*N*,*N*′,*N*′-tetra(*n*-octyl)diglycolamide (TODGA, **3**), and *N*,*N*-didodecyl-2-((1-hexyl-2-oxopiperidin-3-yl)oxy)acetamide (DDHPA, **4**). All three DGAs show comparable nonlinear ascending trends in selectivity from La to Lu; however, they differ in terms of binding affinity for Lns (**2** > **3** > **4**). The stability of DGA–Ln complexes is affected by the size of N-alkyl substituents on the DGA ligand; larger substituents tend to obstruct the metal ion binding site that is decorated with three oxygen donor groups, resulting in diminished affinity for Lns with lower effective nuclear charge.^42,44^ Ligand **5**, with unconstrained amide groups, was synthesized as a control substrate (Figure 3).

### Separation Experiments

The ability of **1** and DGA to separate Lns is evaluated using a two-component, immiscible solvent system: nitric acid (aqueous phase containing **1**) and *n*-dodecane with 10 vol % 1-octanol (oil phase containing DGA). The amount of Ln partitioned from the aqueous phase into the oil phase is measured using inductively coupled plasma optical emission spectroscopy or mass spectrometry. The Ln concentration in each phase is then used to calculate the separation efficiency. The percentage of Lns in aqueous, and the oil phase is presented in Figure 2 for each DGA in the absence and presence of the aqueous complexant **1** (*E* = ([Ln_1_]/[Ln_1_]_0_) × 100%). The distribution ratios (i.e., *D* = [Ln_1_]_org_/[Ln_1_]_aq_) for select Lns are listed in Table 1, which also include selectivities (i.e., SF_Ln_1_/Ln_2__ = *D*_Ln_1__/*D*_Ln_2__), for several Ln pairs (see also Tables S2 and S3). An efficient separation of Lns is achieved when *D*_Ln_1__ > 1 and *D*_Ln_2__ < 1 or *E*_Ln_1__ > 50% and *E*_Ln_2__ < 50%. The separation of 14 Lns (0.5 mM each, 7 mM total concentration) using 0.1 M **2**–**4** alone is shown in Figure 2a–c. Ligand **2** (DMDODGA) nearly quantitatively extracts all Lns from the aqueous phase into the oil phase. Ligand **3** (TODGA) shows reduced affinity for light Lns, which is further pronounced using ligand **4** (DDHPA). Neither of the three DGAs can separate Lns in a single extraction stage. The introduction of water-soluble ligand **1** (3 mM) in the aqueous phase results in lower partitioning of light Lns to the oil phase containing **2**, **3**, or **4** (Figure 2d,e), dramatically changing the selectivity trends across the Ln series. The results show that **1** preferentially binds light Lns and that the extent of Lns retained in the aqueous phase correlates with the change in affinity of DGA for trivalent Lns. The higher concentration of **1** in the aqueous phase (13 mM, Figure 2g–i) further amplifies the selectivity between light and heavy Lns. This two-ligand system yields an efficient separation of light from heavy Lns in a single extraction stage; for example, La–Ce is separated from Tb–Lu (Figure 2h) and La–Nd from Ho–Lu (Figure 2i). Furthermore, an improved separation is demonstrated among the mid-lanthanides. For example, the combination of **1** and **4** yields an improved selectivity in separating adjacent Lns Gd and Tb (SF_Tb/Gd_ = 5.8, Table 1). The selectivity of 15.6 is observed when separating Sm and Nd using **1** and **3**. This system offers a potential strategy to separate radioactive *Pm* from Sm and Nd. The combination of **2** and **1** outperforms the TODGA–DOODA(C2)^26^ and TDdDGA–DOODA(C2)^27^ systems and results in 20 and nearly 2 times higher selectivity for Gd over La, respectively (Table 1). Increased concentration of **1** (25 mM) results in minimal change separating Lns (Table 1), suggesting that the system is at its limit and **1** no longer can or has the ability to outcompete DGA in complexing heavier Lns.

**Table 1:** Distribution Ratios and Separation Factors for Selected Lns

	[1], mM	*D*_La_^a^	*D*_Nd_^a^	*D*_Sm_^a^	*D*_Gd_^a^	SF_Nd/Pr_^a^	SF_Sm/Nd_^a^	SF_Tb/Gd_^a^	SF_Dy/Nd_^a^	SF_Gd/La_^a^
2	0	71	558	1795	2531	2.1	3.2	1.1	4.7	36
	3	0.59	11.0	222	1192	4.0	20	2.0	352	2010
	13	0.28	10.7	68.1	552	4.2	6.4	3.0	451	1992
	25	0.23	2.34	42.2	346	3.2	18	3.0	1313	1512
3	0	2.52	18.6	120	386	2.2	6.4	2.7	70	153
	3	0.21	2.18	28.1	164	2.7	13	3.4	408	783
	13	0.05	0.15	2.35	19.3	2.2	16	5.4	1052	413
	25	0.05	0.14	1.82	14.2	1.8	13	5.3	782	308
4	0	0.18	0.87	6.21	23.0	2.0	7.2	3.3	130	128
	3	0.12	0.32	2.53	13.4	1.8	8.0	3.7	260	109
	13	0.08	0.08	0.16	0.98	0.9	2.0	5.1	133	12
	25	0.06	0.07	0.17	0.88	1.0	2.5	5.8	123	14
3	13^b^	2.65	21.4	163	360	2.3	7.6	1.6	60	136

a Average value of 3 experiments; a full list of *D* ratios and associated errors are summarized in Table S2. *D* = distribution value; *D*_Ln_1__ = [Ln_1_]_org_/[Ln_2_]_aq_. SF = separation factor; SF_Ln_1_/Ln_2__ = *D*_Ln_1__/*D*_Ln_2__.

b Ligand **5** was used instead of ligand **1**.

Like **1**, ligand **5** shows excellent aqueous solubility. However, its performance in separating Lns is very different. As depicted in Figure 3, even at 13 mM concentration, **5** shows limited affinity for light Lns, which is attributed to the larger reorganization energy required to complex with Ln in contrast to a preorganized ligand **1**. Consistently with other reports on 1,10-phenanthroline-2,9-diamides,^64,65^**5** shows preference for light Lns. This is likely due to the rigid core and preorganization of two N donors. On the other hand, 1,10-phenanthroline-2,9-dicarboxylic acid shows the opposite trend, favoring heavier Lns (stability constant log *K*_La_ < log *K*_Gd_),^66^ whereas log *K*_La_ = log *K*_Lu_ using 1,10-phenanthroline-2,9-dicarboxamide.^67^

**Figure 4:**
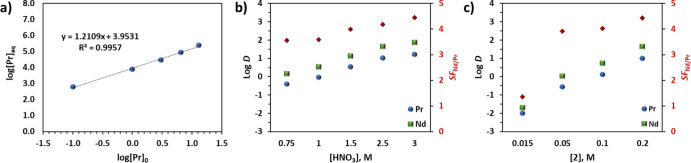
(a) Variation in [Pr]_aq_ after extraction with 0.1 M **2** in *n*-dodecane with 10 vol % 1-octanol from 1 M HNO_3_ containing 13 mM **1** and varying concentrations of Pr ([Pr]_0_). (b) Variation in log *D*_Pr_ and log *D*_Nd_ with aqueous phase acidity after extraction with 0.1 M **2** in *n*-dodecane with 10 vol % 1-octanol from _aq_HNO_3_ containing 13 mM **1**. (c) Variation in log *D*_Pr_ and log *D*_Nd_ after extraction with 15–200 mM **2** in *n*-dodecane with 10 vol % 1-octanol from 1 M HNO_3_ containing 13 mM **1**.

The synergistic use of **1** and **2** was further optimized to maximize the separation of one adjacent Ln pair Nd–Pr using the percentage of Ln extracted as a guide (see the Supporting Information Section 4). The separation of Nd and Pr reaches equilibrium in less than 1 h, and the *D* values for both increase at higher HNO_3_ and **2** concentrations (Figure 4b,c). **2** being a neutral ligand requires coextraction of counterions (i.e., three NO_3_^–^) with Ln; thus, the extraction of Lns with DGA is favored at high nitrate concentration, and the process is reversed at low anion concentration. The increase in log *D* at a higher concentration of **2** indicates that **2** has high affinity for all Ln(III) and that improved separation of Lns can be attained either by lowering its concentration or by increasing that of **1**. The highest selectivity of 4.9 ± 0.2 for Nd–Pr pair was obtained when separating 2.5 mM Nd and Pr, each using 13 mM **1** in 1 M HNO_3_ with 0.1 M **2** in the oil phase and equilibrating for 30 min at 25.5 °C. Next, the recovery of Nd and Pr from each phase after extraction using the **1**–**2**-HNO_3_ system was investigated. Near-quantitative recovery of Nd and Pr from the spent oil phase was obtained after 3 consecutive contacts with an equal volume of deionized water (Figure S5). The dissociation of Pr and Nd from the complexes with **1** in the aqueous phase was promoted by the addition of an equal volume of saturated ammonium carbonate, which resulted in the precipitation of Ln carbonate salts. The charged ligand (CO_3_^2–^) outcompetes neutral ligand **1** in complexing with Ln ions;^68^ furthermore, the thermodynamics of the Ln_2_(CO_3_)_3_ formation is highly favorable due to high crystal lattice energy of the salt. The identity of the precipitate was confirmed using Fourier-transform infrared spectroscopy (FTIR), and the results are in good agreement with commercially available Pr and Nd carbonate salts (Figure S7). Ligand **1** remains in the aqueous solution that can be further recycled in the Ln separation process.

Based on our previous study, the BLPhen family of ligands bind light Lns and the platinum-group metal palladium and reject competing elements such as iron and copper.^51^ The same selectivity profile for d-block metal ions is expected for _aq_BLPhen **1**.

### Spectroscopic Studies

**Figure 5:**
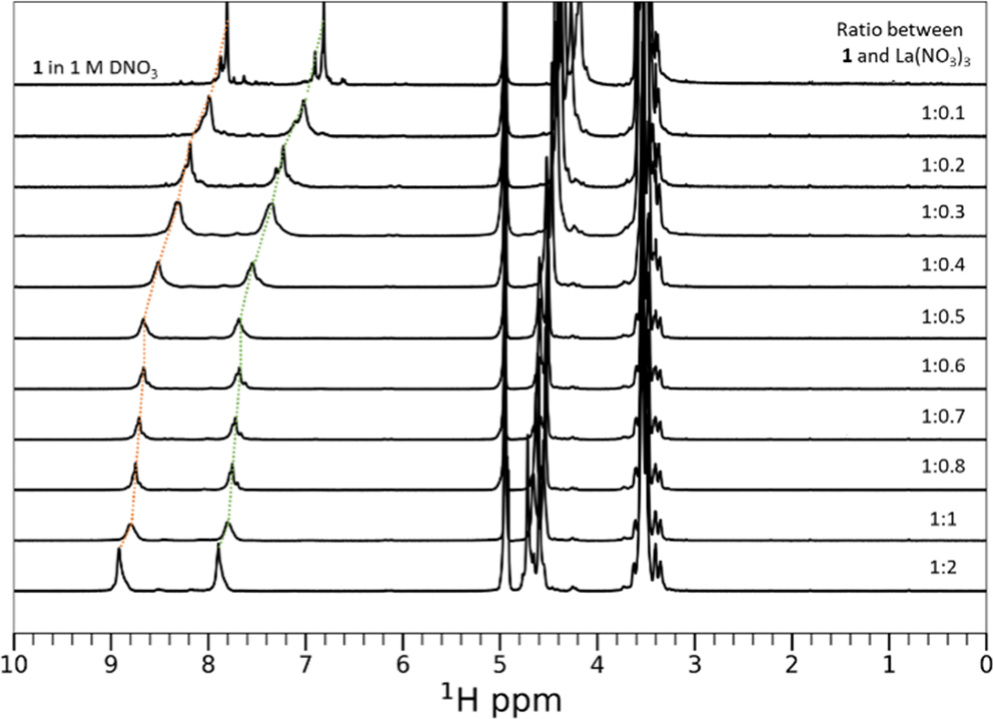
Overlay of ^1^H NMR spectra for **1** in 1 M DNO_3_ in D_2_O without and with increasing amounts of La(NO_3_)_3_ (top to bottom).

Complexation of **1** with Lns was further assessed using nuclear magnetic resonance (NMR) spectroscopy. Figure 5 shows the ^1^H NMR data for **1** with various concentration ratios of La. As the concentration of La increases (top to bottom), a downfield shift of ∼1.1 ppm occurs in the most downfield aromatic ^1^H of **1**, confirming complex formation. In addition, as the concentration of La increases, the spectral lines become increasingly uniform and narrow, suggesting the formation of well-defined species. For **1** alone, some heterogeneity and broadening in the peak structure is observed, which we attribute to aggregate formation. While the ethylene glycol units solubilize **1**, the hydrophobic cores still cluster together to form aggregate structures (vide infra). As the La concentration increases, it disrupts these clusters and stabilizes **1**-La units. The change in chemical shift of the most downfield aromatic ^1^H was used to assess binding constants for La complexes with **1** in 1 M DNO_3_. The results were fitted using the EQNMR^69^ software to yield binding constants of *K*_1_ > 10^2^ M^–1^ and *K*_2_ ≫ 10^5^ M^–1^ for the 1:1 and 2:1 ligands to metal binding modes, respectively. A more significant ∼3 ppm chemical shift change was observed for Pr (Figure S10), which is likely due to differences in the magnitude of the pseudo-contact shifts; however, the narrowing with increased Pr concentration was similar, suggestive of a well-defined chelation. In the Lu titration (Figure S11), some downfield shift was observed, but the peaks became increasingly heterogenous and broad. We attribute this to poor binding affinity where transient interactions occur, but no stable structure was formed, which was consistent with the selectivity data presented above.

**Figure 6:**
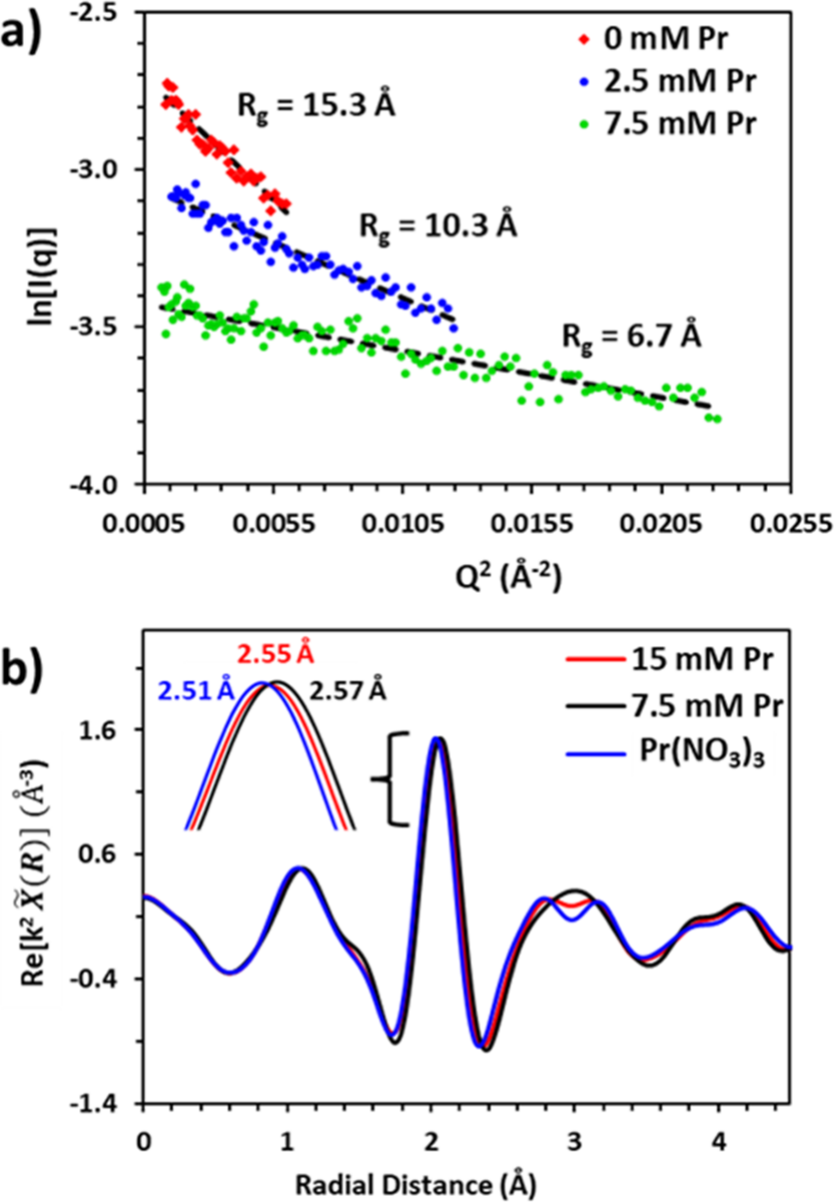
(a) Guinier analysis derived from SAXS measurements at low *q* with linear regression to describe the radius of gyration (*R*_g_). (b) Fourier-transformed EXAFS measurements of various aqueous Pr solutions with 13 mM **1** (red and black traces) and without **1** (blue trace). The inset illustrates that the slight shift in average 1st shell bond distance when Pr is complexed with **1**.

The mesoscale structure of **1** and **1**–Pr complexes in the aqueous solution and the local coordination environment around Pr in **1**–Pr structure were investigated using small-angle X-ray scattering (SAXS) and extended X-ray absorption fine structure (EXAFS) spectroscopy at the Pr L3 edge, respectively (Figures S16 and S17). In the absence of Pr, 13 mM **1** in 1 M HNO_3_ forms large aggregates with a radius of gyration (*R*_g_) of ∼15 Å, as represented by the steepest slope in Figure 6a. The addition of smaller amounts of Pr (2.5 mM) reduces the aggregate size in solution by ∼5 Å.

Under conditions where **1** is expected to be fully coordinated with Pr (assuming the formation of [Pr(**1**)_2_(NO_3_)_3_], when [Pr] = 7.5 mM), the aggregates further reduce in size (*R*_g_ = 6.7 Å). This is consistent with the trends seen in the NMR data above. In addition, variable temperature NMR was taken of **1** without and with Pr (Figure S13). At elevated temperatures (up to 313 K), the spectral quality of **1** degrades dramatically and is irreversible—indicative of additional aggregate formation in solution. In the presence of Pr, however, the ^1^H chemical shifts representing **1** remain unchanged and the peaks narrow due to increased molecular motion. The trend is reversible, suggesting that the complexed species are stable.

The EXAFS measurements provide an element-specific probe of Pr complexation with **1** in 1 M HNO_3_. In this experiment, the Pr aqua complex in the aqueous phase was used as a control (blue trace, Figure 6b). Upon introduction of **1** in the Pr solution, a clear change in the average 1st coordination shell bond distance between Pr and nitrogen/oxygen donor atoms in **1** is observed. Through fitting the EXAFS data to an individual Pr–O scattering path, we find that the average 1st shell bond distance increases by 0.06 and 0.04 Å under conditions where 2:1 (black line) or 1:1 (red line) complexes exist between 13 mM **1** and Pr (7.5 and 15 mM) (Table S1). The calculated distances are likely an average of both Pr–O and Pr–N photoelectron scattering due to the restricted *k*-window of the Pr L3-edge (2.4–9.2 Å^–1^) and consistent with previously measured crystal structures on analogous Pr–BLPhen complexes.^52^ Furthermore, our complementary density functional theory (DFT) calculations indicate a slight shortening of the inner-shell average bond distance on going from 2:1 to 1:1 ligand/metal complexation at higher Pr loading (Figure S18), which is in agreement with the speciation description obtained from our EXAFS results. These observations in addition to the slope analysis (Figure 4a) suggest that 1:1 and 2:1 **1**-Pr complexes readily form in solution, consistent with improved selectivity of **1** toward light lanthanides.

## Conclusions

In conclusion, an efficient and selective lanthanide separation was demonstrated using a two-ligand system. The synergistic interplay between neutral lipophilic and hydrophilic ligands with opposing Ln selectivity led to unprecedented separation profiles for adjacent Lns. Furthermore, the separation of specific Ln pairs can be achieved by selecting a DGA with optimal affinity. The solution structure investigations using solvent extraction and SAXS, NMR, and EXAFS spectroscopies provided useful insights into the speciation of Ln-_aq_BLPhen complexes. The high affinity of **1** for light Lns does not hamper their recovery; however, further optimization is needed to develop a continuous Ln separation cycle. Our future research will focus on designing new hydrophilic and lipophilic ligands with optimal binding affinities to magnify adjacent Ln selectivity and a system that can operate in a closed separation loop.

## Methods

### Synthesis

For the synthesis of 3,4,9,10-tetrakis((2-hydroxyethoxy)methyl)-2,11-dihydrodipyrido[3,4-*b*:4′,3′-*j*]^1,10^ phenanthroline-1,12-dione (_aq_BLPhen, **1**), a solution of *N*^2^,*N*^9^-bis(pivaloyloxy)-1,10-phenanthroline-2,9-dicarboxamide (SI-1, 5 g, 10.7 mmol, 1.0 equiv), 2,2′-(but-2-yne-1,4-diylbis(oxy))bis(ethan-1-ol) (3.6 mL, 23.5 mmol), CsOAc (8.2 g, 42.8 mmol), and Rh catalyst (4 mol %, 265 mg) in MeOH (55 mL) was heated at 55 °C for 24 h. Afterward, the reaction mixture was allowed to cool to room temperature and EtOAc was added to precipitate the crude product. The precipitate was filtered and washed with EtOAc, CH_2_Cl_2_, and Et_2_O. The dried solid was dissolved in minimal DI water and transferred to a pre-column loaded with Celite. The product was purified on a CombiFlash R_f_ automated flash chromatography system using reverse phase RediSepR_f_ Gold C18 Aq 450-g column as a stationary phase and gradient 0–60% (5–15 min) MeOH in H_2_O as an eluent. The product was obtained as a yellow solid (3.5 g, 53% yield). ^1^H NMR (400 MHz, TFA-*d*_1_): δ 9.58 (s, 2H), 8.34 (s, 2H), 5.04 (s, 2H), 5.02 (s, 4H), 4.15–3.87 (m, 18H). ^13^C NMR (100.67 MHz, D_2_O): δ 164.3, 140.6, 135.2, 134.4, 132.8, 131.3, 118.2, 115.3, 112.3, 75.3, 74.6, 68.8, 63.7. HR-MS C_30_H_34_N_4_O_10_ ([M + H]^+^, *m*/*z*): 611.2357 (exp.), 611.2348 (calcd).

### Separation Experiments

General procedure: a 500 microliter (μL) aqueous phase consisting of 7 mM Ln(III) (0.5 mM of each Ln(III)) in 1 M HNO_3_ without or with _aq_BLPhen **1** (3, 13, or 25 mM) was contacted with an equal volume of organic phase containing 0.1 M DGA (**2**, **3**, or **4**). The two phases were contacted in a 1:1 ratio of organic/aqueous by end-over-end rotation in individual 1.8 mL capacity snap-top Eppendorf tubes using a rotating wheel in an airbox set at 25.5 ± 0.5 °C. Contacts were performed in triplicate with a contact time of 1 h. The samples were centrifuged at 1811*g* for 2 min at room temperature to separate the phases. Each triplicate was then subsampled, using a 300 μL aliquot of the aqueous phase transferred to individual polypropylene tubes and diluted with 2% HNO_3_ for analysis using ICP-MS or ICP-OES. Two samples of the initial lanthanide solution were similarly prepared.

### Spectroscopic Studies

Dried La(NO_3_)_3_ was dissolved in 1 M DNO_3_/D_2_O to prepare a 100 mM stock solution. _aq_BLPhen **1** was dissolved in 1 M DNO_3_/D_2_O to prepare a 30 mM stock solution. To 0.5 mL of 30 mM **1** in 1 M DNO_3_/D_2_O were added a specific volume (0.015–0.30 mL) of 100 mM La(NO_3_)_3_ in 1 M DNO_3_/D_2_O, and the solution was further diluted with 1 M DNO_3_/D_2_O to 1.00 mL total volume. The freshly prepared samples were then analyzed using ^1^H NMR spectroscopy. The results were fitted using the EQNMR software to predict binding constants.

SAXS measurements were performed on aqueous samples containing Pr-**1** complexes dissolved in 1 M HNO_3_ using a Xenocs Xeuss 3.0 SAXS instrument with a Mo radiation source. Samples were placed in 1.5 mm quartz capillaries (0.01 mm wall thickness, Charles Supper) and sealed with epoxy. Samples were scanned between 0.02 and 1.1 Å^–1^ in *q*-space (Figure S16); *q* is the momentum transfer: *q* = 4π sin(θ)/λ, where 2θ is the scattering angle and λ is the incident X-ray wavelength.

X-ray absorption spectroscopy of the Pr^3+^ L3 edge was acquired on the 6-BM beamline at NSLS II. Pr^3+^-containing aqueous solutions were placed into PEEK liquid holders with Kapton windows and sealed with epoxy. Measurements included Pr(NO_3_)_3_ dissolved in 1 M HNO_3_ and Pr(NO_3_)_3_ + **1** dissolved in 1 M HNO_3_ in 1:1 and 1:2 molar quantities. The concentration of Pr^3+^ ranged between 5 and 15 mM in solution and required fluorescence detection using a 4 element Vortex detector. Spectral background removal and normalization of the EXAFS were performed using the ATHENA analysis program in which a cut-off distance (*R*_bkg_) of 1.1 Å was used. First shell bond distances between Pr and O were calculated using model photoelectron paths generated from FEFF 6.0 and were used to fit the experimental *k*_2_-weighted FT-EXAFS data utilizing the ARTEMIS software package.

## Supporting Information Available

Characterization of **1** (NMR, FTIR, UV–vis, MS, and EA); Ln-**1** complexes (NMR, SAXS, EXAFS, and DFT calculations); and extraction results (ICP-MS and ICP-OES) (PDF)
