# Supplementary material for: Size Selective Ligand Tug of War Strategy to Separate Rare Earth Elements
Source: JACS Au. 2023 Jan 25;3(2):584–91. doi: 10.1021/jacsau.2c00671 (PMC9976341; doi:10.1021/jacsau.2c00671)
Supplement: Supplementary file 1 — au2c00671_si_001.pdf [file au2c00671_si_001.pdf]

Size-Selective Ligand Tug of War Strategy to Separate Rare Earth Elements

Katherine R. Johnson,† Darren M. Driscoll,‡ Joshua T. Damron,‡ Alexander S. Ivanov,‡ and Santa Jansone-Popova‡*

†Nuclear Energy and Fuel Cycle Division, Oak Ridge National Laboratory, Oak Ridge, TN 37831, United States

‡Chemical Sciences Division, Oak Ridge National Laboratory, Oak Ridge, TN 37831, United States

Table of contents

1. **Experimental details** 2
   - **General information** 2
   - **Instrumental Methods** 2
2. **Ligand synthesis and characterization** 3
3. **Solvent Extraction Experiments** 6
   - **Extraction of 14 Lns with DGAs in the absence and presence of 1** 8
   - **Separation of Pr(III) and Nd(III) in DMDODGA-1-HNO_3 system** 9
   - **Precipitation of Pr(III) and Nd(III) carbonates** 12
   - **Slope analysis** 13
4. **Characterization of Ln-1 complexes** 14
   - **^{1}H NMR spectroscopy: Complexation titration of 1 with Ln nitrates** 14
   - **SAXS measurements** 18
   - **EXAFS measurements** 19
   - **Density functional theory (DFT) calculations** 20
5. **NMR Spectra** 22
6. **References** 25

1. Experimental details

General information

All commercially obtained reagents were analytical grade and used as received. Dry solvents were purchased as dry. $Ln(NO_3)_3$ (Ln^{III}= La^{III}, Nd^{III}, or Lu^{III}) were prepared by drying in a vacuum oven (80 ^{o}C) overnight. Commercially available compounds were purchased from Aldrich Chemical Co., Acros Organics, Alfa Aesar or TCI America and were used without further purification. DMDODGA and TODGA were supplied by Marshallton Labs and no less than 98 % purity.

Instrumental Methods

Nuclear Magnetic Resonance (NMR) Spectroscopy. NMR 1D spectra were recorded on an Avance III-400 MHz NMR spectrometer (Bruker Company) equipped with a 5 mm BBO probe. For ^{1}H and ^{13}C NMR spectra, the residual solvent peak or sodium formate was used as an internal reference.

Infrared Spectroscopy. All FT-IR spectra were measured on a Perkin Elmer Frontier. The IR data for each sample were collected in the range 4000−600 cm^{-1}, with 32 scans at 1 cm^{-1} resolution per spectrum. A background correction for CO_2 and H_2O was applied.

Mass Spectrometry (MS). High resolution MS analyses were performed under contract by University of Texas Arlington Mass Spectrometric Facility using positive mode.

Elemental Analysis. Analyses were performed under contract by Atlantic Microlab, Inc. (Norcross, GA). Carbon, hydrogen, nitrogen and fluorine analyses were performed on a Perkin-Elmer Model 2400 Series II Auto analyzers or Carlo Erba Model 1108 Analyzers adopting a technique based on a modification of the classical Pregl and Dumas methods.

Absorption Spectroscopy. Absorption spectra were measured on a Varian Cary 4E UV-Vis spectrophotometer equipped with deuterium and tungsten halogen lamps (source changeover set to 350 nm). The absorption spectra were collected using a scan speed of 600 nm/min in the range 200 – 800 nm with a photodiode detector. All spectra were background corrected, using solvent as the blank. Spectra of the ligands and their complexes were collected at 1.5×10^{-4} M in distilled water at 24 ^{o}C.

Inductively Coupled Plasma Mass Spectrometry (ICP-MS). Spent aqueous extraction solutions from extraction experiments containing numerous Ln(III) ions (La(III) – Lu(III, withholding Pm(III)) were analyzed on a ThermoFisher Scientific iCAP TQ spectrometer equipped with a prepFAST M5 autodilution autosampler set to TQ O_2 mode. Calibration curves were collected using at least a 5 point curve using Rare Earth Element Mix (TraceCert, Sigma Aldrich) in a 2% HNO_3 matrix. Sample solutions were measured in triplicate.

Inductively Coupled Plasma Optical Emission Spectroscopy (ICP-OES). Spent aqueous extraction solutions from experiments using [Pr(III) + Nd(III)] were analyzed on a ThermoFisher Scientific iCAP 7400 spectrometer equipped with a prepFAST M5X autodilution autosampler. Calibration curves were collected using at least a 5 point curve using Rare Earth Element Mix (TraceCert, Sigma Aldrich) in a 2% HNO_3 matrix. Sample solutions were measured in triplicate.

2. Ligand synthesis and characterization

The synthesis of DDHPA (**4**)[1], **SI-1**[2] and **SI-5**[3] is described in the literature.

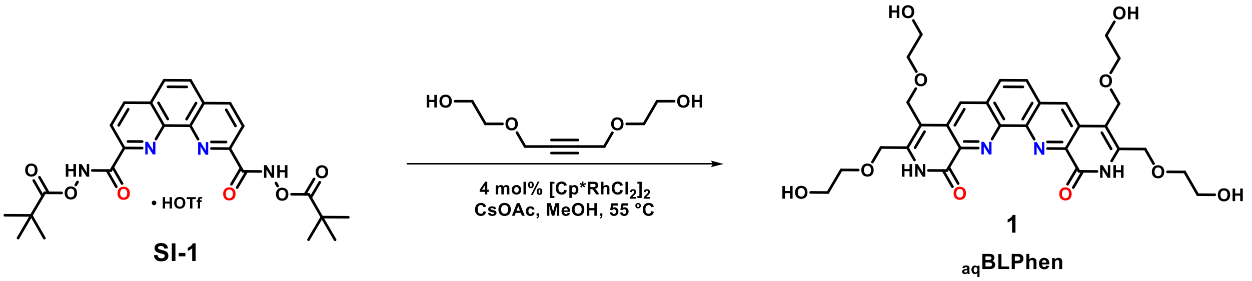

The synthesis of 3,4,9,10-tetrakis((2-hydroxyethoxy)methyl)-2,11-dihydrodipyrido[3,4-b:4',3'-j][1,10]phenanthroline-1,12-dione ($_{aq}$BLPhen, **1**): solution of N^2,N^9-bis(pivaloyloxy)-1,10-phenanthroline-2,9-dicarboxamide (**SI-1**, 2 g, 3.2 mmol, 1.0 equiv.), 2,2'-(but-2-yne-1,4-diylbis(oxy))bis(ethan-1-ol) (1.4 mL, 9.2 mmol), CsOAc (3.3 g, 5.37 mmol), and Rh catalyst (5 mol%, 106 mg) in MeOH (25 mL) was heated at 55 ˚C for 24 hours. Afterwards, the reaction mixture was allowed to cool to room temperature and EtOAc was added to precipitate crude product. The precipitate was filtered, washed with EtOAc, CH_2Cl_2, and Et_2O. The dried solid was dissolved in minimal volume of DI water and transferred to pre-column loaded with Celite®. The product was purified on CombiFlash Rf automated flash chromatography system using reverse phase RediSepRf Gold C18 Aq 150-g column as a stationary phase (note: column was washed with 0.1% TFA solution prior to separation) and gradient 0-60% (5-15 min) MeOH in H_2O as an eluent (product elutes at 14 min). The product was obtained as a TFA salt, as red or dark brown solid (1.1 g, 44% yield). **^{1}H NMR** (400 MHz, TFA-d_1) δ 9.58 (s, 2H), 8.34 (s, 2H), 5.04 (s, 2H), 5.02 (s, 4H), 4.15-3.87 (m, 18H). **^{13}C NMR** (100.67 MHz, D_2O) δ 164.3, 140.6, 135.2, 134.4, 132.8, 131.3, 118.2, 115.3, 112.3, 75.3, 74.6, 68.8, 63.7. **FT-IR** (neat, ν, cm^{-1}): 3600 – 2990 (ν_{max} = 2923, br., O – H), 3020 – 2710 (ν_{max} = 2877 (C – H), 1674 (C = O), 1200 – 1160 (ν_{max} = 1176, C –O – C). **HR-MS** $C_{30}H_{34}N_4O_{10}$ ($[M + H]^+$, m/z): 611.2357 (exp.) 611.2348 (calc.). **EA** calculated for (**1**)•$(CF_3CO_2H)_{1.7}$•$(H_2O)_2$: C, 47.85; H, 4.78; N, 6.70; F, 11.35; found: C, 47.96; H, 4.71; N, 6.75; F, 12.01.

To obtain neutral **1**, crude material was purified on CombiFlash Rf automated flash chromatography system using reverse phase RediSepRf Gold C18 Aq 450-g column as a stationary phase that has never been exposed to dilute TFA solution and gradient 0-60% MeOH in H_2O as an eluent. The product was obtained as a yellow solid (3.5 g, 53% yield).

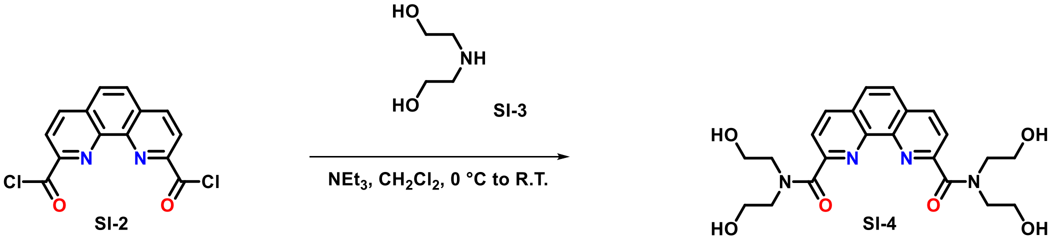

The synthesis of N^2,N^2,N^9,N^9-tetrakis(2-hydroxyethyl)-1,10-phenanthroline-2,9-dicarboxamide (**SI-4**): the solution of diethanolamine (**SI-3**, 1.4 mL, 0.0145 mol) and NEt_3 (2.0 mL, 0.0145 mol) in anhydrous dichloroethane (30 mL) was cooled in ice-water bath before the addition of 1,10-phenanthroline-2,9-dicarbonyl dichloride (2.0 g, 6.55 mmol). The reaction mixture was stirred at 0 ˚C for 30 min, then allowed to warm up to room temperature and stir for 12 hours. The reaction solvent was evaporated, the remaining residue was dissolved in minimum volume of DI water. The product precipitated from this solution. The white solid was collected by filtration,

washed with cold water, then Et_2O and dried under vacuum (1.55 g, 53% yield). **^{1}H NMR** (400 MHz, D_2O) δ 8.91-8.82 (m, 2H), 8.17-8.10 (m, 4H), 3.86 (dt, J = 5.3, 49.3 Hz, 8H), 3.69-3.52 (m, 8H). **^{13}C NMR** (100.67 MHz, DMSO-d_6) δ 168.4, 154.3, 143.0, 137.7, 128.5, 127.2, 123.4, 59.0, 58.5, 51.2, 48.2. **EA** calculated for **SI-4**: C, 59.72; H, 5.92; N, 12.66; found: C, 57.49; H, 5.83; N, 12.13.

Due to the very low solubility of **SI-4** in water and diluted nitric acid, this substrate could not be used as a control in this study. The compound **SI-6** was identified as a more suitable candidate.

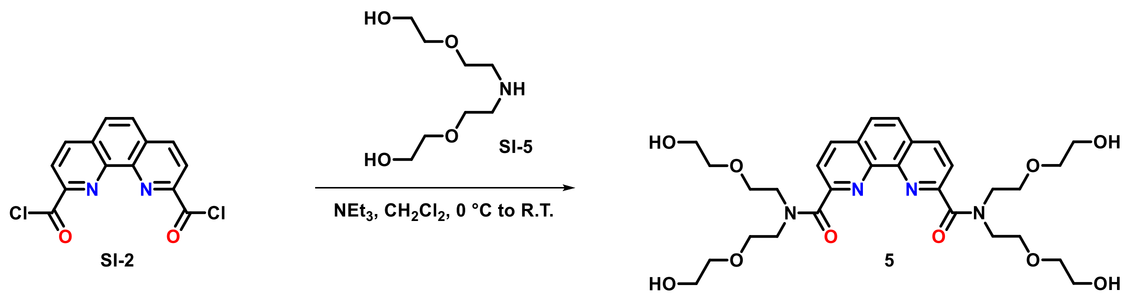

The synthesis of N^2,N^2,N^9,N^9-tetrakis(2-(2-hydroxyethoxy)ethyl)-1,10-phenanthroline-2,9-dicarboxamide (**5**): the solution of bis(hydroxyethoxyethyl)amine (**SI-5**, 2.8 g, 0.0145 mol) and NEt_3 (2.0 mL, 0.0145 mol) in anhydrous dichloroethane (30 mL) was cooled in ice-water bath before the addition of 1,10-phenanthroline-2,9-dicarbonyl dichloride (**SI-2**, 2.0 g, 6.55 mmol). The reaction mixture was stirred at 0 ˚C for 30 min, then allowed to warm up to room temperature and stir for 12 hours. The reaction solvent was evaporated, the remaining residue was dissolved in minimum volume of DI water and transferred to pre-column loaded with Celite®. The product was purified on CombiFlash Rf automated flash chromatography system using reverse phase RediSepRf Gold C18 Aq 150-g column as a stationary phase and gradient 0-50% (2-12 min) MeOH in H_2O as an eluent (product elutes at 12 min). The product was obtained as light beige oil (2.43 g, 60% yield). **^{1}H NMR** (400 MHz, D_2O) δ 8.43-8.33 (m, 2H), 7.90-7.80 (m, 2H), 7.75-7.65 (m, 2H), 4.00-3.87 (m, 8H), 3.87-3.60 (m, 16H), 3.55-3.42 (m, 4H), 3.35-3.22 (m, 4H). **^{13}C NMR** (100.67 MHz, D_2O) δ 174.3, 155.7, 146.2, 141.2, 131.8, 130.1, 125.2, 74.6, 74.4, 70.8, 70.4, 63.3, 63.0, 51.7, 48.4. **FT-IR** (neat, ν, cm^{-1}): 3370 – 3120 (ν_{max} = 3392, br., O – H), 3015 – 2754 (ν_{max} = 2862 (C – H), 1609 (C = O), 1140 – 1090 (ν_{max} = 1124, C –O –C).

Ligand **1** shows good stability in D_2O. In 1 M DNO_3 **1** quite rapidly precipitates, however, when complexed with Ln(III) salt, **1** remains in the solution without undergoing aggregation/degradation (Figure S1).

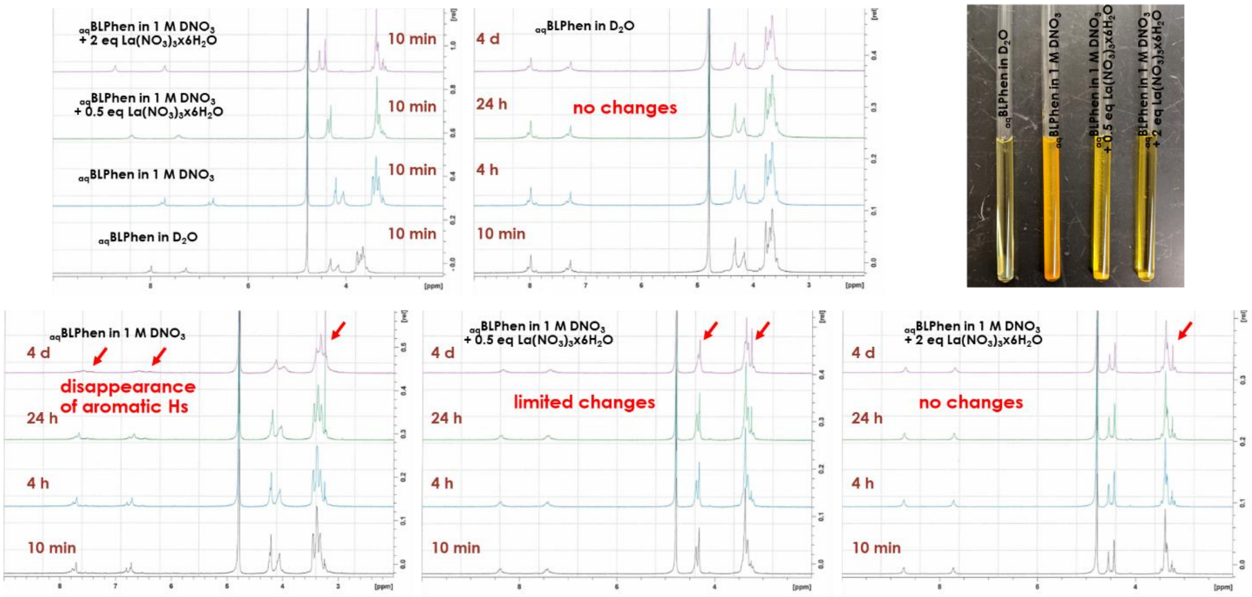

Figure S1. Stability of **1** in D_2O, 1 M DNO_3, 1 M DNO_3 with 0.5 equiv. $La(NO_3)_3$, and 1 M DNO_3 with 2 equiv. $La(NO_3)_3$.

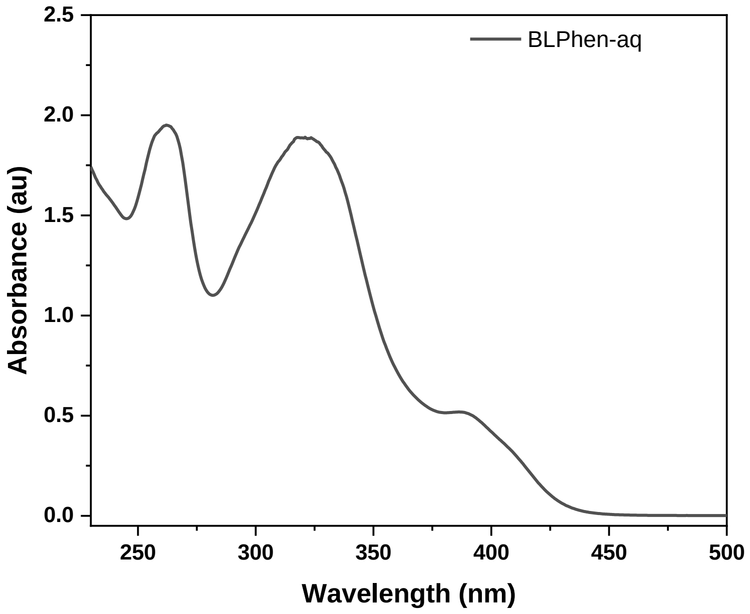

Figure S2. Absorbance spectrum of **1** in DI water at 25.5 ^{o}C; [**1**] = 0.15 mM in water.

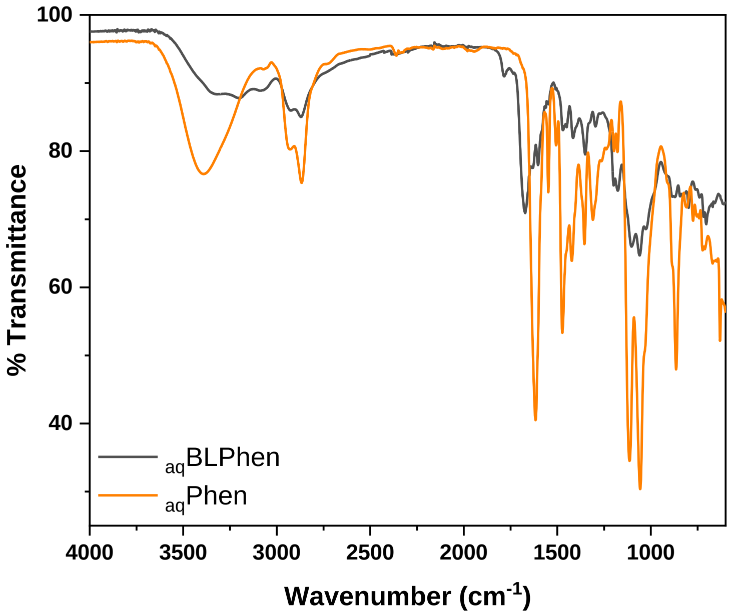

Figure S3 FT-IR spectrum of **1** and **5**.

3. Solvent Extraction Experiments

All reagents were of analytical grade and used without further purification. The aqueous phases were prepared by preparing a stock solution of either 7 mM Ln(III) solution (0.5 mM of La(III) – Lu(III), withholding Pm(III)) or 5 mM bimetallic Ln(III) solution, [Pr(III) + Nd(III)], (2.5 mM each) in 1 M HNO_3 unless noted otherwise. The desired mass of $_{aq}$BLPhen (**1**) was added to an empty volumetric flask and diluted to the mark with the acidic Ln(III) solution and mixed for at least 5 minutes before extraction. The organic phase containing DMDODGA dissolved in *n*-dodecane 10% (v/v) *n*-octanol was preequilibrated with 1 M HNO_3 solution before extraction.

Procedure for extraction with Ln(III). A 500 microliter (μL) aqueous phase consisting of 7 mM Ln(III) (0.5 mM of each Ln(III)) in 1 M HNO_3 was contacted with an equal volume of organic phase containing 0.1 M DGA (DMDODGA, TODGA, or DDHPA). The two phases were contacted at a 1:1 ratio of organic/aqueous by end-over-end rotation in individual 1.8 mL capacity snap-top Eppendorf tubes using a rotating wheel in an airbox set at 25.5 ± 0.5°C. Contacts were performed in triplicate with a contact time of 1 hour. The samples were centrifuged at 1,811 × *g* for two minutes at room temperature to separate the phases. Each triplicate was then subsampled, using a 300 μL aliquot of the aqueous phase transferred to individual polypropylene tubes and diluted with 2% HNO_3 for analysis. Two samples of the initial lanthanide solution were similarly prepared.

General procedure for extraction with [Pr(III) + Nd(III)] solution. A 500 microliter (μL) aqueous phase containing 5 mM [Pr(III) + Nd(III)] (2.5 mM each) in 1 M HNO_3 and was contacted with an equal volume of organic phase containing DMDODGA. The two phases were contacted at a 1:1 ratio of organic/aqueous by end-over-end rotation in individual 1.8 mL capacity snap-top Eppendorf tubes using a rotating wheel in an airbox set at 25.5 ± 0.5°C. Contacts were performed in triplicate. The samples were centrifuged at 1,811 × *g* for two minutes at room temperature to separate the phases. Each triplicate was then subsampled, using a 300 μL aliquot of the aqueous phase transferred to individual polypropylene tubes and diluted with 2% HNO_3 for analysis. Two samples of the initial lanthanide solution were similarly prepared.

The distribution ratio (D) was measured by measuring the concentration of the metal ion in the aqueous phase after extraction and by comparing it to the initial concentration. As such, D values were determined using the following equation (Eq. 2):

$$D_{Ln} = \frac{C_i - C_f}{C_f} \qquad \text{Eq. 2}$$

where C_i and C_f are the concentrations of the metal ions in the aqueous phase before (i = initial) and after (f = final) extraction, respectively.
The separation factor (SF) was determined using the following equation (Eq. 3):

$$SF_{Ln1/Ln2} = \frac{D_{Ln1}}{D_{Ln2}} \qquad \text{Eq. 3}$$

where D_{Ln1} and D_{Ln2} are the distribution ratios of Ln(III).

Extraction of 14 Lns with DGAs in the absence and presence of 1

Table S1. Distribution values for Ln(III) and the corresponding errors below. [b] Ligand **5** used instead of ligand **1**.

		Distribution (D) values														
**Ligand**	**[1], mM**	**La**	**Ce**	**Pr**	**Nd**	**Pm**	**Sm**	**Eu**	**Gd**	**Tb**	**Dy**	**Ho**	**Er**	**Tm**	**Yb**	**Lu**
**2**	**0**	70.99	137.15	264.68	557.65		1794.68	2190.11	2530.55	2875.03	2629.12	2775.54	2551.57	2694.72	2617.30	2731.89
	**3**	0.59	0.99	2.72	10.96		222.05	650.20	1191.85	2392.29	3851.33	4502.78	4366.14	4779.30	4562.75	4843.13
	**13**	0.28	0.44	2.55	10.67		68.08	225.81	551.54	1675.21	4812.62	8241.84	14863.94	15856.18	16324.88	20105.29
	**25**	0.23	0.31	0.72	2.34		42.18	135.87	346.47	1030.03	3072.76	4975.81	9718.38	10367.15	10673.62	10052.14
**3**	**0**	2.52	4.72	8.56	18.58		119.60	267.50	385.55	1025.77	1300.90	1844.42	2397.47	2507.61	2354.57	2953.98
	**3**	0.21	0.44	0.80	2.18		28.07	84.86	164.15	554.65	891.12	1457.99	2118.19	2279.55	2089.15	2699.58
	**13**	0.05	0.05	0.07	0.15		2.35	8.36	19.30	104.36	158.12	431.16	590.59	946.84	627.82	1281.35
	**25**	0.05	0.06	0.08	0.14		1.82	5.93	14.18	75.32	112.99	314.24	441.00	719.86	476.94	1003.40
**4**	**0**	0.18	0.28	0.43	0.87		6.21	14.97	23.02	76.95	112.46	173.18	251.51	295.86	284.41	384.54
	**3**	0.12	0.12	0.18	0.32		2.53	7.14	13.40	49.89	82.66	142.87	220.99	268.41	254.59	354.64
	**13**	0.08	0.08	0.09	0.08		0.16	0.51	0.98	4.97	10.88	26.14	52.94	80.88	74.59	131.90
	**25**	0.06	0.06	0.07	0.07		0.17	0.45	0.88	5.15	8.38	21.41	38.85	60.65	55.12	103.95
**3**	**13b**	2.65	4.82	9.46	21.35		163.19	162.74	360.26	590.95	1281.79	1897.71	2599.34	2990.72	2971.36	2559.73

		error														
**Ligand**	**[1], mM**	**La**	**Ce**	**Pr**	**Nd**	**Pm**	**Sm**	**Eu**	**Gd**	**Tb**	**Dy**	**Ho**	**Er**	**Tm**	**Yb**	**Lu**
**2**	**0**	1.95	4.06	5.71	9.51		46.77	69.64	44.74	52.44	93.93	50.62	133.97	93.38	85.57	91.91
	**3**	0.01	0.03	0.11	0.20		16.13	122.06	334.75	1250.74	2412.00	3071.47	2926.28	3234.93	3063.40	3303.59
	**13**	0.01	0.01	0.08	0.18		2.41	6.99	13.98	17.81	157.31	446.16	1514.52	1615.61	1663.37	2487.51
	**25**	0.01	0.01	0.03	0.01		1.00	4.18	5.26	24.30	64.14	162.64	647.48	690.70	711.11	621.88
**3**	**0**	0.05	0.06	0.13	0.23		1.35	5.48	13.18	77.71	124.43	224.61	348.34	391.86	341.15	501.72
	**3**	0.03	0.05	0.06	0.11		1.23	3.68	6.98	25.05	68.16	146.09	255.15	290.25	283.90	388.14
	**13**	0.01	0.01	0.00	0.00		0.04	0.12	0.51	2.74	10.03	44.23	90.06	175.49	102.08	296.58
	**25**	0.01	0.00	0.01	0.00		0.01	0.09	0.20	1.56	3.38	20.53	43.04	83.17	44.59	136.92
**4**	**0**	0.02	0.02	0.02	0.03		0.13	0.42	0.38	2.04	3.30	6.31	12.77	14.91	14.46	21.92
	**3**	0.04	0.04	0.04	0.04		0.11	0.35	0.51	1.42	2.64	5.67	6.24	9.34	5.85	6.91
	**13**	0.00	0.01	0.01	0.01		0.02	0.02	0.04	0.13	0.36	0.78	2.21	3.35	2.65	5.22
	**25**	0.02	0.00	0.01	0.01		0.02	0.01	0.02	0.06	0.30	1.18	1.45	1.57	2.12	2.16

Table S2. Separation factors. [b] Ligand **5** used instead of ligand **1**.

		Separation Factors (SF)							
**Ligand**	**[1], mM**	**Lu/La**	**Nd/Pr**	**Eu/Sm**	**Gd/Eu**	**Tb/Gd**	**Sm/Nd**	**Dy/Nd**	**Gd/La**
**2**	**0**	38.5	2.1	1.2	1.2	1.1	3.2	4.7	35.6
	**3**	8168.9	4.0	2.9	1.8	2.0	20.3	351.5	2010.3
	**13**	72607.9	4.2	3.3	2.4	3.0	6.4	450.9	1991.8
	**25**	43871.2	3.2	3.2	2.6	3.0	18.0	1313.2	1512.1
**3**	**0**	1170.9	2.2	2.2	1.4	2.7	6.4	70.0	152.8
	**3**	12884.4	2.7	3.0	1.9	3.4	12.8	407.9	783.4
	**13**	27405.8	2.2	3.6	2.3	5.4	15.6	1052.0	412.8
	**25**	21781.3	1.8	3.3	2.4	5.3	12.6	782.4	307.9
**4**	**0**	2141.3	2.0	2.4	1.5	3.3	7.2	129.9	128.2
	**3**	2893.6	1.8	2.8	1.9	3.7	8.0	259.9	109.4
	**13**	1667.1	0.9	3.2	1.9	5.1	2.0	132.7	12.4
	**25**	1615.6	1.0	2.6	2.0	5.8	2.5	123.0	13.7
**2**	**13b**	965.3	2.3	1.0	2.2	1.6	7.6	60.0	135.9

Separation of Pr(III) and Nd(III) in DMDODGA-1-HNO_3 system

The percent of extracted Ln(III) (%E) was determined by using the following equation (Eq. 4):

$$\%E_{Ln} = \frac{C_i - C_f}{C_i} \times 100 \qquad \text{Eq. 4}$$

The percent recovery of Ln(III) from the loaded organic phase can be determined using the following equation (Eq. 5):

$$\%R = \frac{C_{strip}}{C_i} \times 100 \qquad \text{Eq. 5}$$

where C_{strip} is the concentration of the metal ions in the aqueous phase after a single strip cycle.

Stripping procedure. An equal volume of deionized water (500 mL) was added to the loaded organic phase and was contacted by end-over-end rotation in individual 1.8 mL capacity snap-top Eppendorf tubes using a rotating wheel in an airbox set at 25.5 ± 0.5°C. Contacts were performed in triplicate. The samples were centrifuged at 1,811 × *g* for two minutes at room temperature to separate the phases. Each triplicate was then subsampled, using a 400 μL aliquot of the aqueous phase transferred to individual polypropylene tubes and diluted with 2% HNO_3 for analysis.

Table S3. Distribution ratio (D_{Ln}) and separation factors ($SF_{Nd/Pr}$) determined for the extraction of Nd(III) and Pr(III) with various concentrations of DMDODGA using $_{aq}$BLPhen as a holdback agent. Aqueous phase: [$_{aq}$BLPhen] = 13 mM, [HNO_3] = 1 M; Organic phase: [DMDODGA] = 15 – 200 mM in n-dodecane 10% v/v 1-octanol.

[DMDODGA], M	D_{Pr}	D_{Nd}	$SF_{Nd/Pr}$
0.015	0.01	0.02	1.36
0.05	0.27	1.06	3.91
0.1	1.34	5.41	4.03
0.2	9.83	43.50	4.43

Table S4. Distribution ratio (D_{Ln}) and separation factors ($SF_{Nd/Pr}$) determined for the extraction of Nd(III) and Pr(III) with DMDODGA using $_{aq}$BLPhen as a holdback agent at different contact times. Aqueous phase: [$_{aq}$BLPhen] = 13 mM, [HNO_3] = 1.0 M; Organic phase: [DMDODGA] = 15 – 200 mM in n-dodecane 10% v/v 1-octanol.

minute(s)	D_{Pr}	D_{Nd}	$SF_{Nd/Pr}$
1	1.878	5.690	3.03 ± 0.6
10	1.810	6.665	3.68 ± 0.08
30	1.786	6.896	3.87 ± 0.01
60	1.632	6.673	4.13 ± 0.16
120	1.719	6.502	3.78 ± 0.06
300	1.697	6.420	3.78 ± 0.08

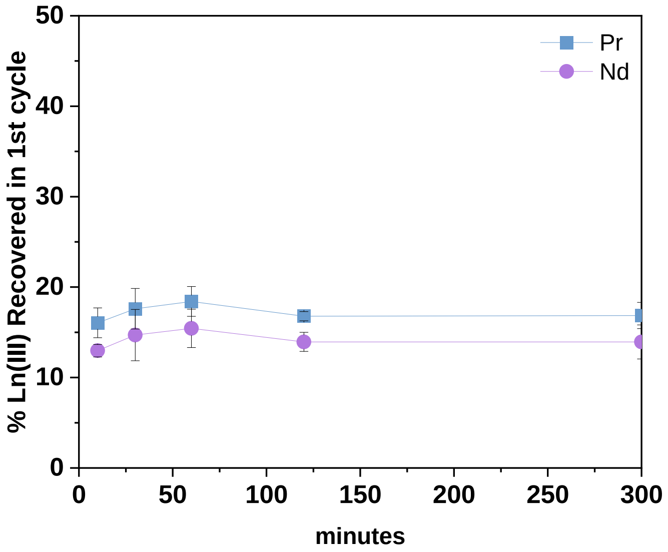

Figure S4. Effect of stripping time (minutes) on the percent Nd(III) recovered from a loaded organic phase by deionized water. O:A = 1:1.

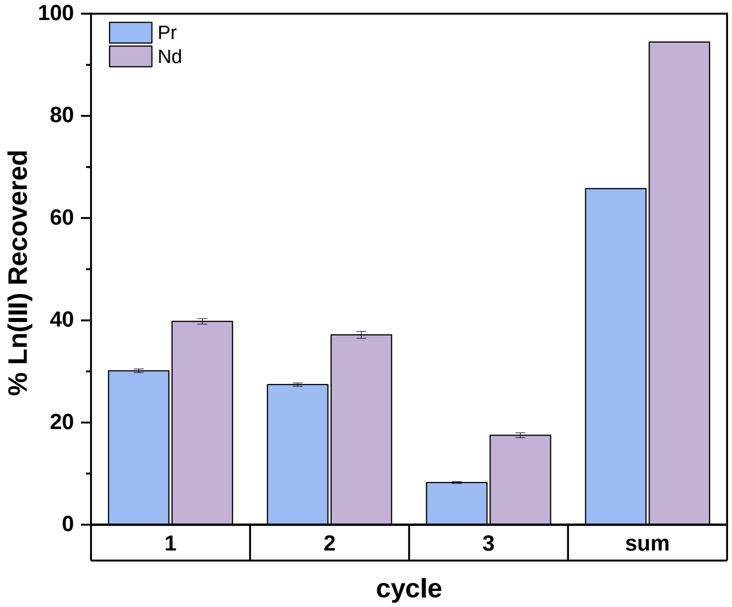

Figure S5. Number of stripping cycles contacted with the loaded organic phase using deionized water. Conditions: O:A = 1:1, 25.5 ± 0.5 °C, contacted for 30 minutes.

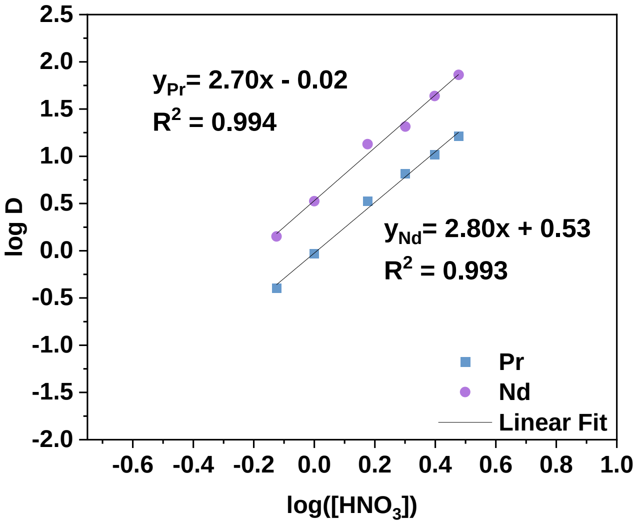

Figure S6. Effect of nitrate ion concentration (M) on distribution ratio. Aqueous phase: [$_{aq}$BLPhen] = 13 mM, [HNO3] = 0.75 – 3 M; Organic phase: [DMDODGA] = 100 mM in n-dodecane 10% v/v 1-octanol.

Table S5. Distribution ratio (D_{Ln}) and separation factors ($SF_{Nd/Pr}$) determined for the extraction of Nd(III) and Pr(III) with DMDODGA using $_{aq}$BLPhen as a holdback agent at various concentrations of nitrate ions. Aqueous phase: [$_{aq}$BLPhen] = 13 mM, [HNO_3] = 0.75 – 3 M; Organic phase: [DMDODGA] = 100 mM in n-dodecane 10% v/v 1-octanol.

[HNO3], M	D_{Pr}	D_{Nd}	$SF_{Nd/Pr}$
0.75	0.40	1.42	3.55
1	1.34	5.41	4.03
1.5	3.37	13.44	3.99
2	5.03	20.59	4.09
2.5	10.41	43.45	4.17
3	16.40	72.83	4.44

Precipitation of Pr(III) and Nd(III) carbonates

Precipitation procedure. An equal volume of aqueous solution containing $_{aq}$BLPhen (15 mM) and [Pr(III) + Nd(III)] (5 mM) in 1 M HNO_3 was added dropwise to a saturated ammonium carbonate solution in duplicate. The samples were left in the fridge for 2 days, then centrifuged at 1,811 × *g* for two minutes at room temperature. The solution was decanted and filtered. 300 mL aliquots of each solution were prepared in 2% HNO3 solution for ICP-OES analysis. The remaining solid was washed with water several times, dried under reduced pressure, and analyzed using powder XRD and FT-IR. Control experiments containing no $_{aq}$BLPhen were also tested following this procedure and used for comparison

The percent of metals precipitated from the stripping solutions can be determined using the following equation (Eq. 6):

$$\%P = \frac{C_i - C_f}{C_i} \times 100 \qquad \text{Eq. 6}$$

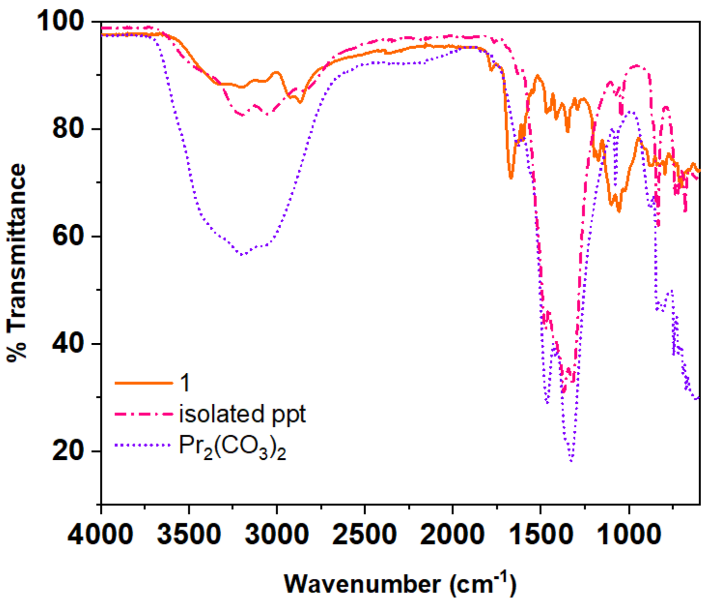

Figure S7. FT-IR spectra of precipitate obtained after reacting aqueous solution containing 5 mM of Pr(III) and **1** with saturated $(NH_4)_2CO_3$ solution (1:1 v/v; pink dash dotted trace), commercially available Pr(III) carbonate (purple dotted trace), and **1** (solid orange trace).

Slope analysis

A 500 uL aqueous phase containing **1** (13mM) and different concentrations of Pr in 1 M HNO_3 was contacted with 500 uL of a preequilibrated solution of DMDODGA (0.1 M). The phases were rotated for 1 h in an airbox set to 25 °C. The samples were centrifuged for 2 minutes then the phases separated. 375 uL of the aqueous phase was transferred to 4.2 mL of 2% HNO_3 for ICP-MS analysis.

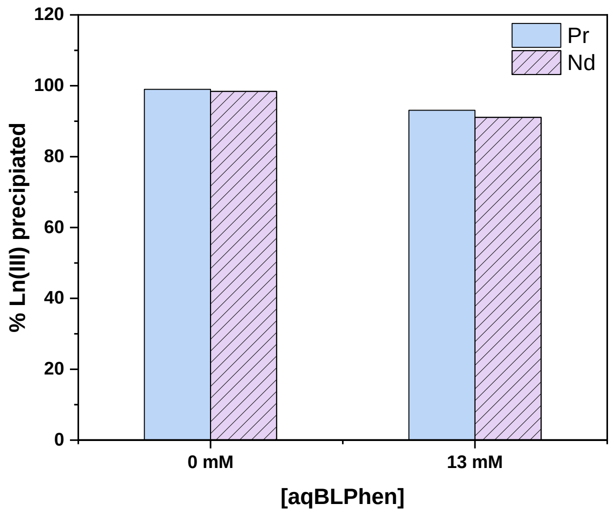

Figure S8. Percent of Ln(III) precipitated (%P) from a solution containing 13 mM $_{aq}$BLPhen in 1M HNO_3. Values in parentheses indicate %P when $_{aq}$BLPhen is not present in solution.

4. Characterization of Ln-1 complexes

^{1}H NMR spectroscopy: Complexation titration of 1 with Ln nitrates

Sample Preparation. Dried Ln(III) nitrate salts (Ln(III) = La(III), Pr(III), or Lu(III)) were dissolved in 1 M DNO_3/D_2O to prepare a 71 mM Ln(III) stock solution. $_{aq}$BLPhen (**1**, 30 mM) was also dissolved in 1M DNO_3/D_2O to prepare a 30 mM stock solution. Ln(III) complexes were prepared in situ by mixing the appropriate volumes of stock solutions for at least 24 h at room temperature before collecting the following spectra.

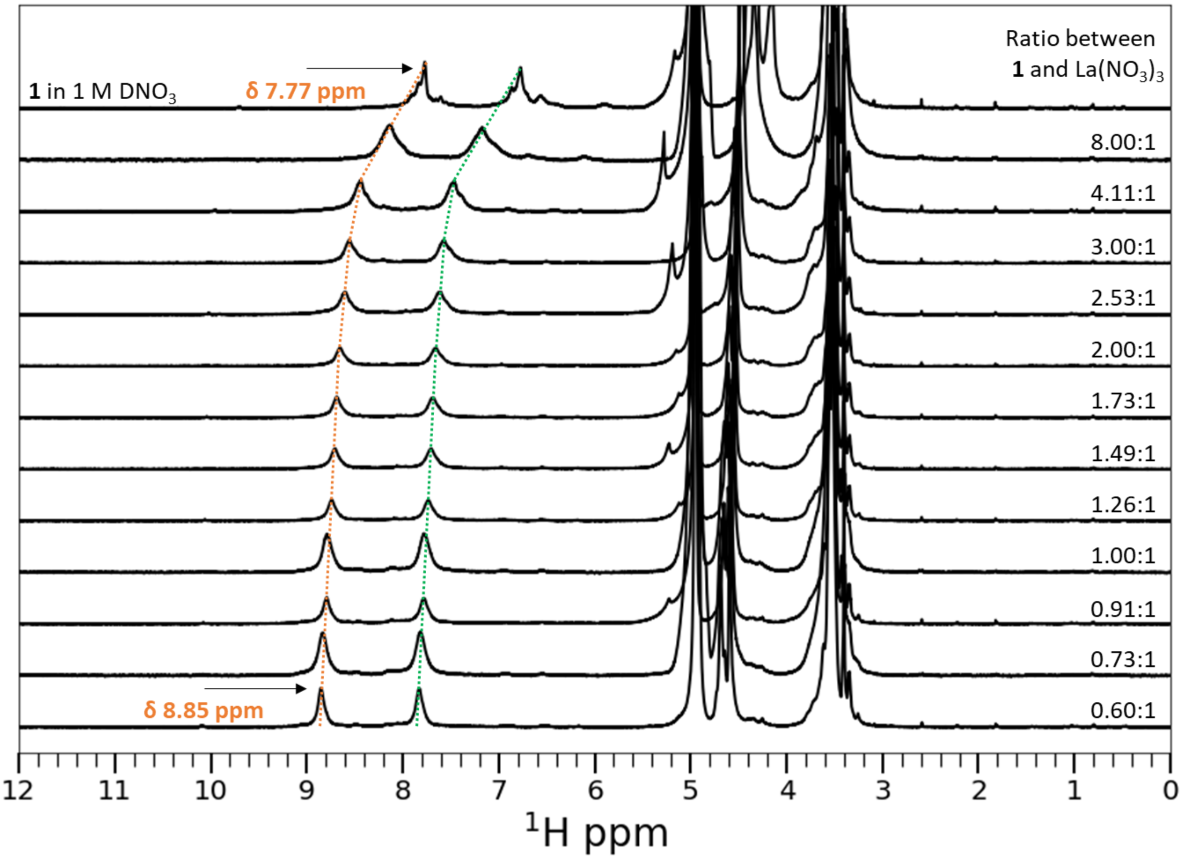

Figure S9. ^{1}H NMR spectra of **1** titrated with La(III). Concentration ratios shown on the right.

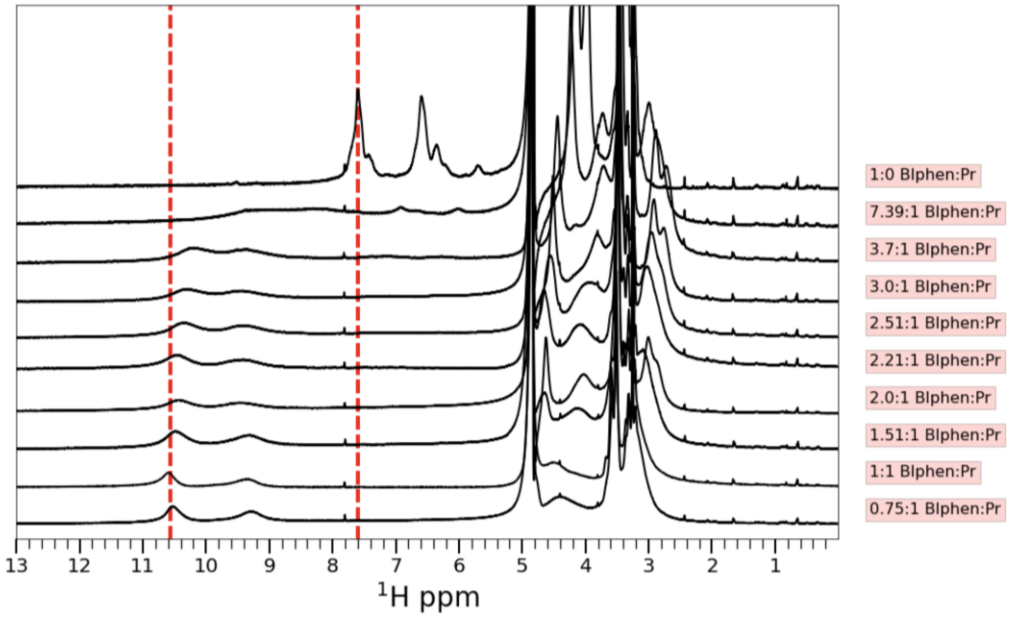

Figure S10. ^{1}H NMR spectra of **1** titrated with Pr(III). Concentration ratios shown on the right.

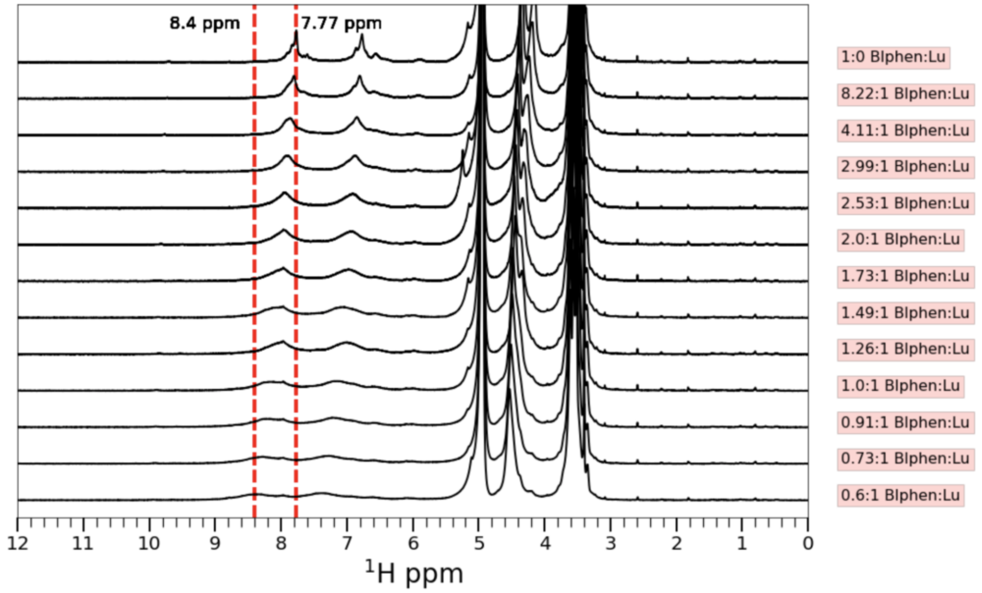

Figure S11. ^{1}H NMR spectra of **1** titrated with Lu(III). Concentration ratios shown on the right.

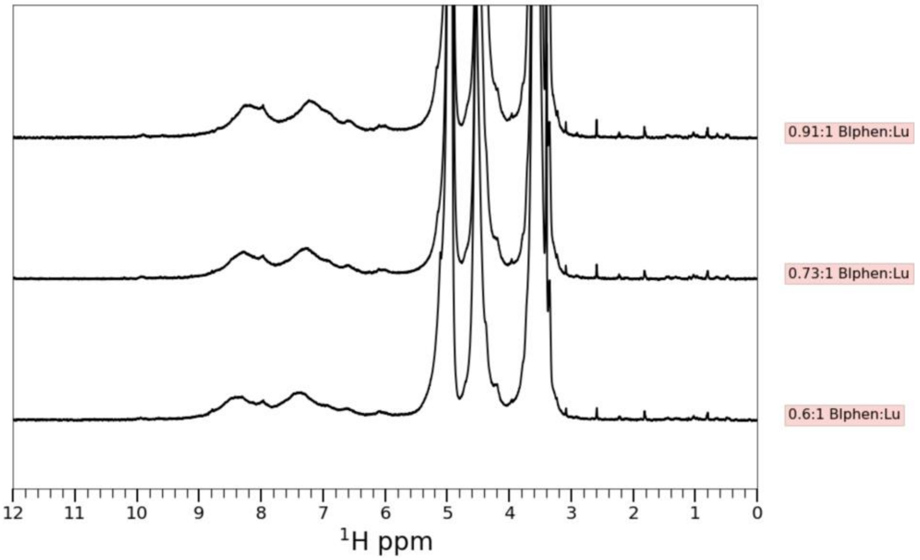

Figure S12. ^{1}H spectra of **1** with high concentrations of Lu(III). Note the broad, inhomogeneous nature of the peaks suggesting multiple, poorly defined aggregate species.

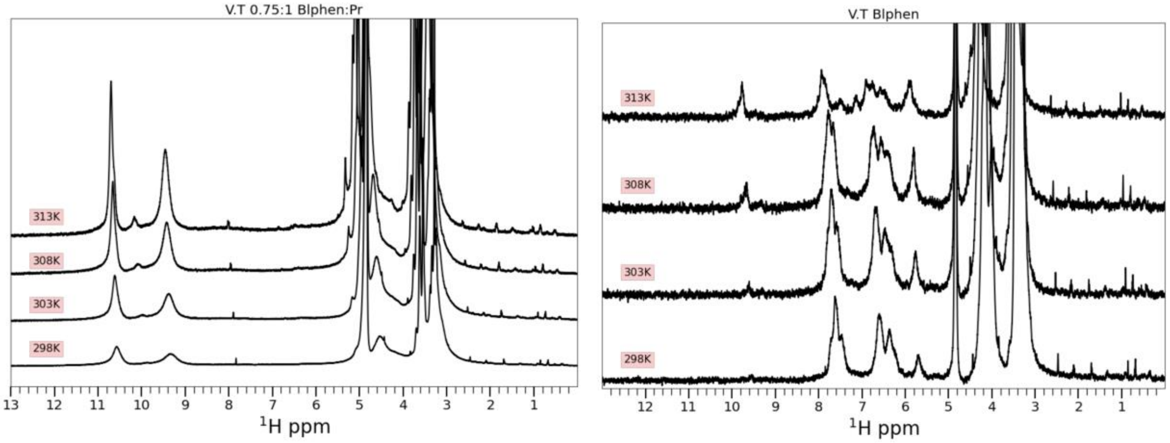

Figure S13. Left: Variable temperature data of ^{1}H NMR spectra of **1** titrated with Pr(III) at 0.75:1 **1**:Pr ratio. Right: Variable temperature data of **1** alone. Clearly, the Pr(III) titrated system is much more stable than the **1** alone. Severe aggregation in NMR tube was observed for **1** alone.

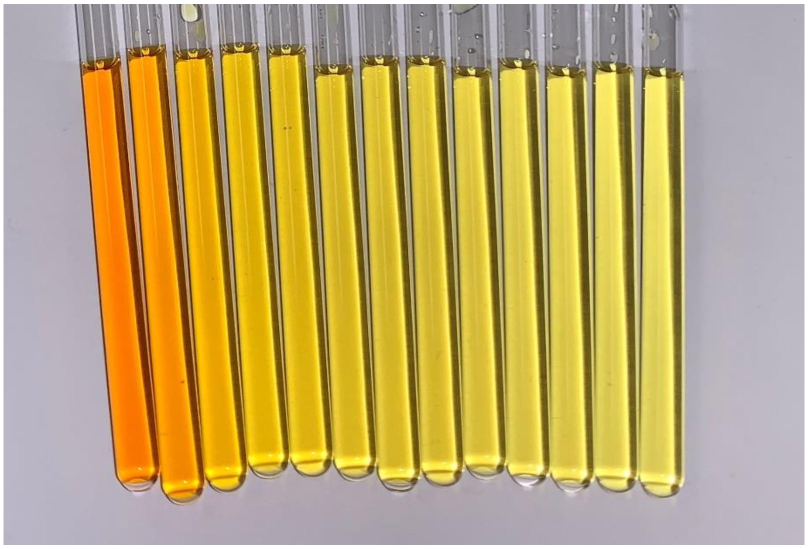

Figure S14. Photograph of **1** (left) and $La(NO_3)_3$:**1** samples with varying ligand to metal stoichiometries. [**1**] = 13 mM in 1 M DNO_3/D_2O. The L:M ratio from left to right is 1:0, 8.22:1, 4.11:1, 2.99:1, 2.53:1, 2.00:1, 1.73:1, 1.49:1, 1.26:1, 1.00:1, 0.91:1, 0.73:1, 0.60:1.

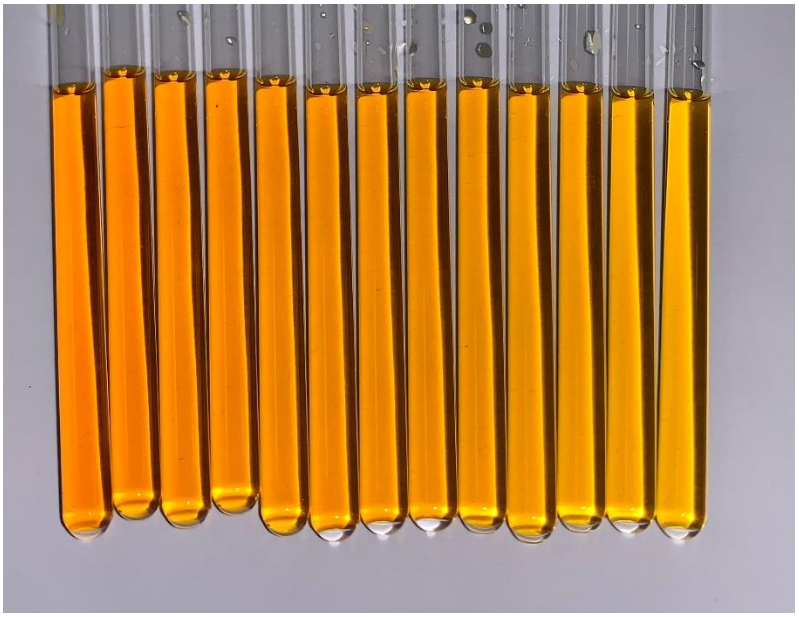

Figure S15. Photograph of **1** (left) and $Lu(NO_3)_3$:**1** samples with varying ligand to metal stoichiometries. [**1**] = 13 mM in 1 M DNO_3/D_2O. The L:M ratio from left to right is 1:0, 8.22:1, 4.11:1, 2.99:1, 2.53:1, 2.00:1, 1.73:1, 1.49:1, 1.26:1, 1.00:1, 0.91:1, 0.73:1, 0.60:1.

Sample Preparation. Dried $La(NO_3)_3$ was dissolved in 1 M DNO_3/D_2O to prepare a 100 mM stock solution. $_{aq}$BLPhen **1** (neutral, not the TFA salt) was dissolved in 1 M DNO_3/D_2O to prepare a 30 mM stock solution. To 0.5 mL of 30 mM **1** in 1 M DNO_3/D_2O were added a specific volume (0.015-0.30 mL) of 100 mM $La(NO_3)_3$ in 1 M DNO_3/D_2O and the solution was further diluted with 1 M DNO_3/D_2O to 1.00 mL total volume. The freshly prepared samples were then analyzed using ^{1}H NMR spectroscopy.

Exp. #	1, equiv.	La, equiv.	V of La stock, mL	c of La, mM	V of 1 stock, mL	V of 1 M DNO_3/D_2O	δ Ar-H1, ppm	δ Ar-H2, ppm
**1**	1	0.1	0.015	1.5	0.5	0.485	7.9925	7.0180
**2**	1	0.2	0.030	3.0	0.5	0.470	8.1872	7.2267
**3**	1	0.3	0.045	4.5	0.5	0.455	8.3272	7.3411
**4**	1	0.4	0.060	6.0	0.5	0.440	8.5154	7.5472
**5**	1	0.5	0.075	7.5	0.5	0.425	8.6698	7.6867
**6**	1	0.6	0.090	9.0	0.5	0.410	8.6633	7.6796
**7**	1	0.7	0.105	10.5	0.5	0.395	8.7111	7.7214
**8**	1	0.8	0.120	12.0	0.5	0.380	8.7476	7.7527
**9**	1	1.0	0.150	15.0	0.5	0.350	8.7933	7.8044
**10**	1	2.0	0.300	30.0	0.5	0.200	8.9178	7.8945
						**δ Ar-H1, ppm (without La):**	7.8051	6.8118

The results were fitted using EQNMR software to predict binding constants.

```
Calculations by WinEQNMR2 Version 2.00 by Michael J. Hynes
Program run at 13:00:51   on 06/22/2022

 Practical data for L, LM and L2M system.
 Reactions: L + M = LM(beta1 = logK1); 2L + M = L2M (beta2 = logK2)
 15 mM of L was titrated with 30 mM of M
 File prepared by Santa, June 8 2022

  Equilibrium constants are log10 values

NO.  A   PARAMETER    DELTA     ERROR    CONDITION   DESCRIPTION
   1  1  2.54832E+00 5.000E-02 1.326E+00 7.477E+02 BETA1
   2  1  6.23329E+00 5.000E-02 8.886E-01 4.603E+01 BETA2
   3  1  7.82011E+00 1.000E-02 4.596E-02 3.423E+00 L SHIFT
   4  1  9.80383E+00 2.000E-02 2.958E+00 8.652E+02 LA SHIFT
   5  1  8.70857E+00 2.000E-02 9.651E-02 5.930E+01 L2A SHIFT

0RMS ERROR = 2.83E-02  MAX ERROR = 4.56E-02 AT OBS.NO.  5
 RESIDUALS SQUARED = 4.00E-03
 RFACTOR =     0.2338 PERCENT

 NO. A   EXPT. DEL  CALC. DEL     RESIDUAL     % DEV   WEIGHT     Anion         Ligand        pH
   1 1  7.9925E+00  7.9973E+00 -4.7736E-03 -5.9726E-02  1.0000E+00  1.5000E-03  1.5000E-02  0.0000E+00
   2 1  8.1872E+00  8.1737E+00  1.3466E-02  1.6447E-01  1.0000E+00  3.0000E-03  1.5000E-02  0.0000E+00
   3 1  8.3272E+00  8.3473E+00 -2.0095E-02 -2.4132E-01  1.0000E+00  4.5000E-03  1.5000E-02  0.0000E+00
   4 1  8.5154E+00  8.5078E+00  7.5579E-03  8.8755E-02  1.0000E+00  6.0000E-03  1.5000E-02  0.0000E+00
   5 1  8.6698E+00  8.6242E+00  4.5631E-02  5.2633E-01  1.0000E+00  7.5000E-03  1.5000E-02  0.0000E+00
   6 1  8.6633E+00  8.6894E+00 -2.6116E-02 -3.0146E-01  1.0000E+00  9.0000E-03  1.5000E-02  0.0000E+00
   7 1  8.7111E+00  8.7286E+00 -1.7452E-02 -2.0034E-01  1.0000E+00  1.0500E-02  1.5000E-02  0.0000E+00
   8 1  8.7476E+00  8.7564E+00 -8.7757E-03 -1.0032E-01  1.0000E+00  1.2000E-02  1.5000E-02  0.0000E+00
   9 1  8.7933E+00  8.7964E+00 -3.0937E-03 -3.5183E-02  1.0000E+00  1.5000E-02  1.5000E-02  0.0000E+00
  10 1  8.9178E+00  8.9043E+00  1.3491E-02  1.5128E-01  1.0000E+00  3.0000E-02  1.5000E-02  0.0000E+00

 TOLERANCE ON SUM OF SQUARES    0.0100
 TOLERANCE ON EIGEN VALUES    0.0001
 CONVERGANCE AFTER   19  ITERATIONS
```

SAXS measurements

Small-angle X-ray scattering (SAXS) measurements were performed on aqueous samples containing Pr–**1** complexes dissolved in 1 M HNO_3 using a Xenocs Xeuss 3.0 SAXS instrument with a Mo radiation source. Samples were placed into 1.5 mm quartz capillaries (0.01 mm wall thickness, Charles Supper) and sealed with epoxy. Samples were scanned between 0.02 – 1.1

Å^{-1} in q-space (Figure S16); q is the momentum transfer: q=4π sin(θ)/λ, where 2θ is the scattering angle and λ is the incident X-ray wavelength. Solvent-subtracted SAXS measurements depict a clear change in aggregate structure in the q-range between 0.03 – 0.7 Å. Aqueous solutions containing **1** and 1 M HNO_3 with no added Pr^{3+} show an uptick of scattering intensity and increase in slope at low-q compared with solutions containing Pr^{3+} (red and blue traces, Figure S10). This suggests larger aggregate structure formed in the aqueous ligand **1** solution. On the other hand, the slope at low-q (≈ 0.03 Å^{-1}) is relatively flat for the 7.5 mM Pr^{3+} solution in which the majority of **1** (13 mM) is expected to be complexed with Pr^{3+} and is consistent with little supramolecular aggregate-aggregate interactions.

Futher interpretation of the SAXS data employed Guinier's approximation at low-q scattering (Eq. 1):[4]

$$I(q) \approx I(0)e^{\frac{-q^2R_g^2}{3}} \qquad \text{Eq.1}$$

Where R_g is the radius of gyration and I(0) is the intensity at zero scattering angle (q = 0). A linear fit of the ln(I) vs. q^2 at low values of q ($q_{max} \times R_g < 1.3$ for spherical particles) provides a non-structural analysis for the size of the scattering species within the sample.

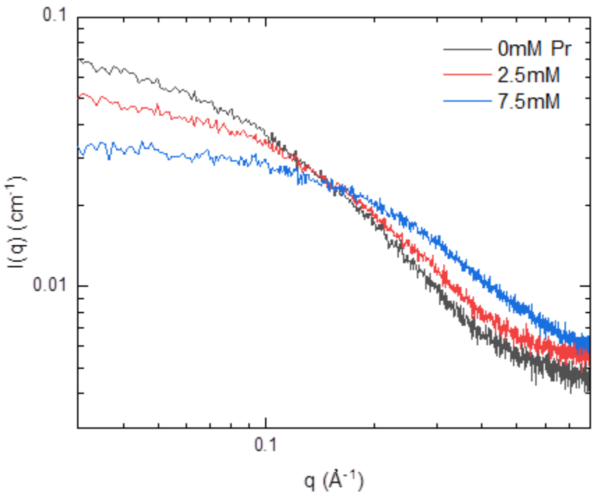

Figure S16. Solvent subtracted SAXS measurements of various **1** solutions.

EXAFS measurements

X-ray absorption spectroscopy of the Pr^{3+} L3-edge was acquired on the 6-BM beamline at NSLS II. Pr^{3+}-containing aqueous solutions were placed into PEEK liquid holders with Kapton windows and sealed with epoxy. Measurements included $Pr(NO_3)_3$ dissolved in 1 M HNO_3 and $Pr(NO_3)_3$ + **1** dissolved in 1 M HNO_3 in 1:1 and 1:2 molar quantities. The concentration of Pr^{3+} ranged between 5 – 15 mM in solution and required fluorescence detection using a 4 element Vortex detector. Spectral background removal and normalization of the EXAFS was performed using the ATHENA analysis program in which a cut-off distance (R_{bkg}) of 1.1 Å was used.[5] First shell bond distances between Pr & O were calculated using model photoelectron paths generated from FEFF 6.0 and

used to fit the experimental k^2-weighted FT-EXAFS data utilizing the ARTEMIS software package.[5a]

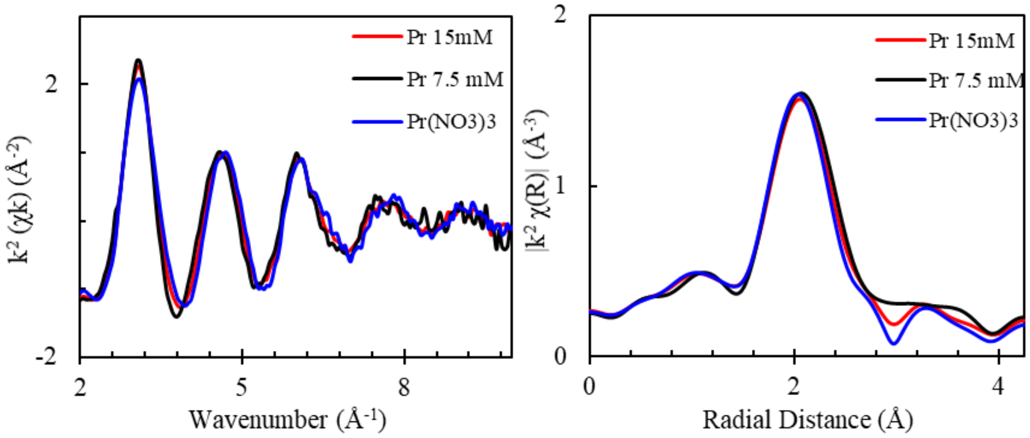

Figure S17. k^2-weighted EXAFS data of various aqueous Pr^{3+} solutions.

The 5 mM $Pr(NO_3)_3$ solution dissolved in 1 M HNO_3 was utilized to mimic Pr^{3+} in a fully aqua coordinated first shell environment. A first shell fit of the $Pr(NO_3)_3$ solution utilizing a 4 parameters fit (coordination number, Debye-Waller factor, path length and energy shift of the photoelectron) found a similar average Pr – O bond distance (2.51 ± 0.02 Å) to reported literature values (2.507)for fully aqua coordinated Pr^{3+}.[6] A similar fitting treatment was used for the additional two Pr^{3+} samples (15 mM and 7.5 mM Pr) containing **1** and found average Pr – O bond distances of 2.55 ± 0.01 and 2.57 ± 0.01 Å, respectively. Based on the k-window used in the fitting (2.4 – 9.2 $Å^{-1}$) for the Pr^{3+} + **1** solutions, the first shell oscillation is likely an average of both the Pr – O and Pr – N photoelectron scattering correlations found in solution. Hence, the average first shell bond distance for solutions containing Pr–**1** complexes shown in the EXAFS is found to be longer compared to the Pr–aqua complexes, which is consistent with the trend observed for the corresponding crystal structures (Table S6).[2] Additionally, the lack of pronounced radial features found beyond the 1st coordination shell (beyond 3 Å in Figure S17) suggest that the Pr–**1** coordinated structure is fluctional in aqueous solution and EXAFS correlations pertaining to the 2nd coordination shell are likely dampened. Therefore, fitting to individual scattering paths to model Pr:**1** beyond the 1st shell structure is unattainable

Table S6. Calculated average Pr 1st shell bond distances from EXAFS measurements

**Sample**	**R (Å)**
5mM $Pr(NO_3)_3$	2.51 ± 0.02
7.5 mM $Pr(NO_3)_3$ + 13 mM ***1***	2.57 ± 0.01
15 mM $Pr(NO_3)_3$ + 13 mM ***1***	2.54 ± 0.01

Density functional theory (DFT) calculations

DFT calculations were carried out with the Gaussian 16, Revision A.03 software package[7] using the BP86 functional[8], which was previously shown to reproduce the experimental coordination structures of the related lanthanide complexes with ligands containing O/N donor groups[9], and in the current study it was used in conjunction with the D3 version of Grimme dispersion[10],

employing the original D3 damping function to account for the van der Waals interactions. Standard 6-31+G(d) basis set was adopted for main-group elements and hydrogen for geometry optimization. Pr was modeled using the large-core (LC) relativistic effective core potential (RECP) and the associated basis set.[11] Since LC RECP calculations include the 4f electrons in the core, they were done on a pseudo-singlet-state configuration. Frequency calculations were performed at the BP86-D3/LC/6-31+G(d) to ensure that optimized geometries were minima on the potential energy surface. Several configurations were considered for 1:1 and 2:1 $_{aq}$BLPhen:Pr complexes in an effort to find the most energetically stable structure. To reduce the computational demand in our search for the most stable configuration, the long alkyl substituents present in $_{aq}$BLPhen were shortened to the hydroxymethyl groups. Nitrate counterions were not explicitly considered in the computational models since they are expected to be fully dissociated from lanthanide complexes in aqueous environment at dilute metal ion concentrations.[12] Therefore, for the 1:1 complex Pr coordination sphere was saturated with additional explicit water molecules. The most stable structures are shown in Figure S18 along with their corresponding inner-shell average bond distances. As can be seen, the DFT results for the 1:1 aqueous complex are in good agreement with the EXAFS data [first sphere average distance: 2.558 Å (DFT) vs. 2.55 Å (EXAFS)], consistent with the presence of the 1:1 $_{aq}$BLPhen:Pr complex at 15 mM Pr loading. When the concentration of Pr is low (7.5 mM), there is a slight shift in the average distance according to the EXAFS (2.57 Å), signifying possible presence of 2:1 species, which might form in solution along with the 1:1 complex. This is also consistent with the DFT structure of the 2:1 complex, exhibiting a somewhat longer (2.587 Å) inner-shell bond distance as compared to the 1:1 coordination.

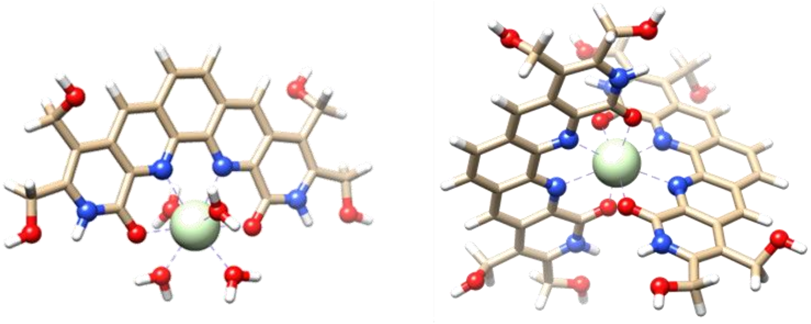

$Pr(BLPhen)^{3+}$
avg. inner-shell distance:
2.558 Å

$Pr(BLPhen)_2^{3+}$
avg. inner-shell distance:
2.587 Å

Figure S18. DFT optimized structures of 1:1 and 2:1 $_{aq}$BLPhen:Pr complexes at the BP86-D3/LC/6-31+G(d) level. Color scheme: Pr, tea green; O, red; N, blue; C, beige; H, white.

5. NMR Spectra

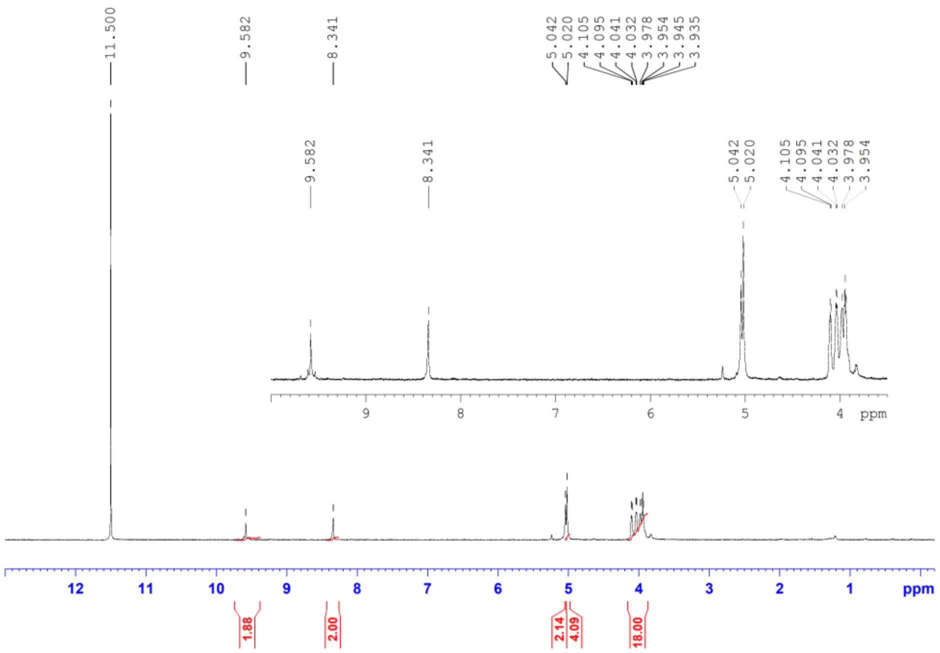

Figure 19. ^{1}H NMR spectrum of **1** in TFA-d_1.

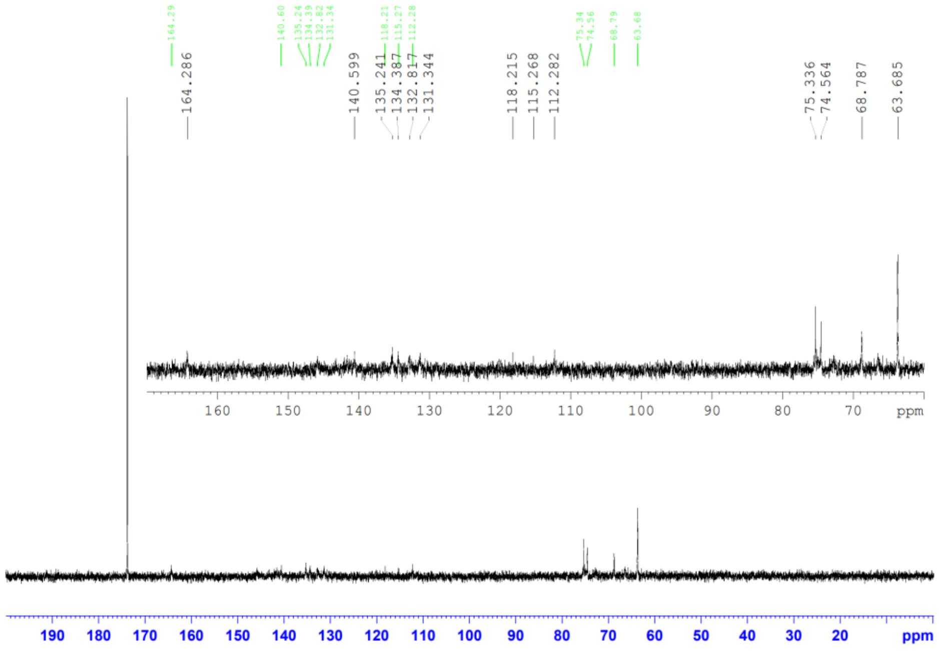

Figure S20. ^{13}C NMR spectrum of **1** in D_2O with $NaHCO_2$ as an internal standard.

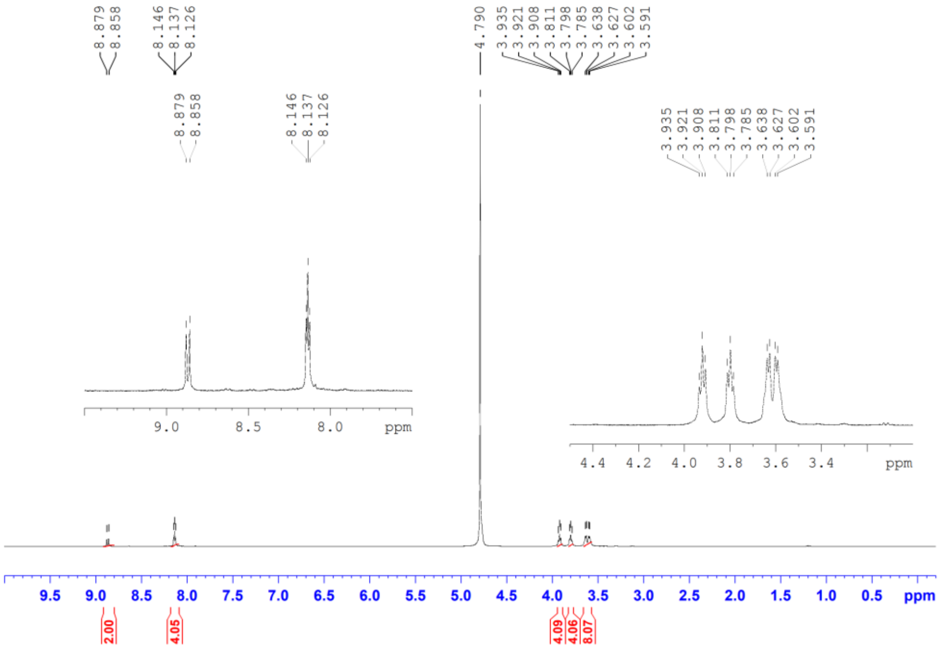

Figure S21. ^{1}H NMR spectrum of **SI-4** in D_2O.

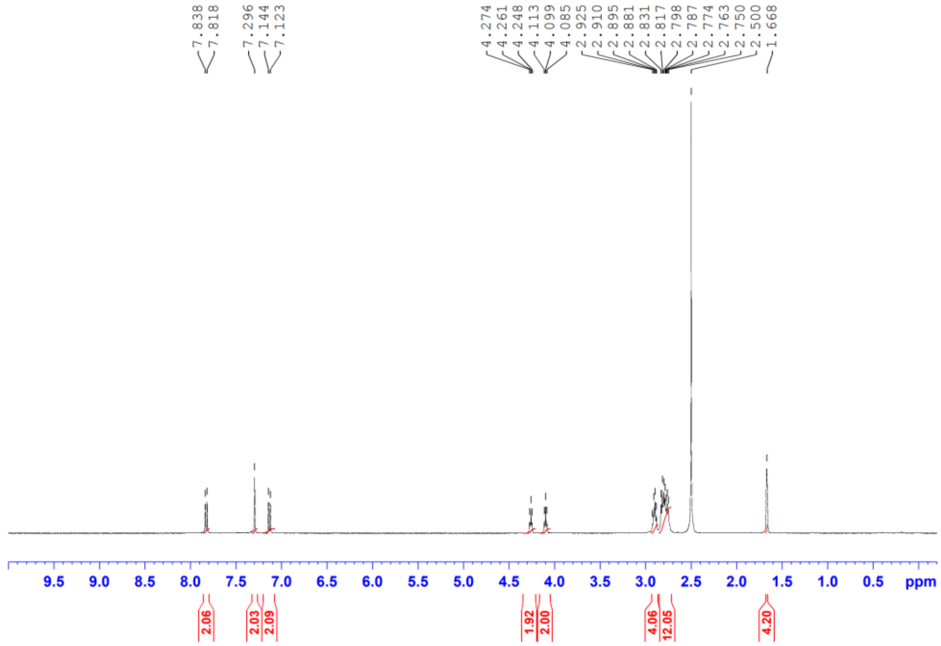

Figure S22. ^{1}H NMR spectrum of **SI-4** in DMSO-d_6.

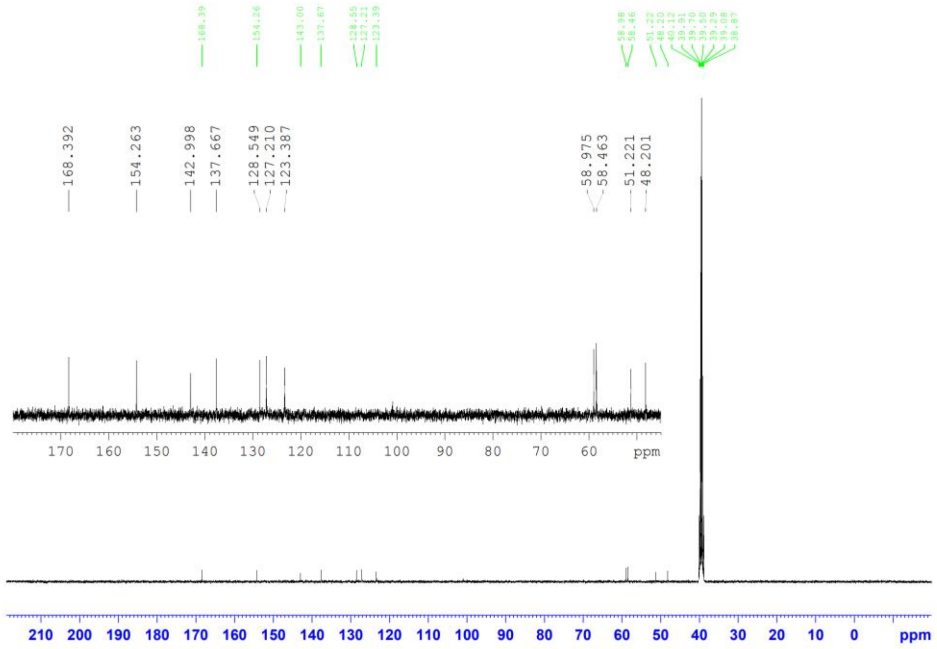

Figure S23. ^{13}C NMR spectrum of **SI-4** in D_2O in DMSO-d_6.

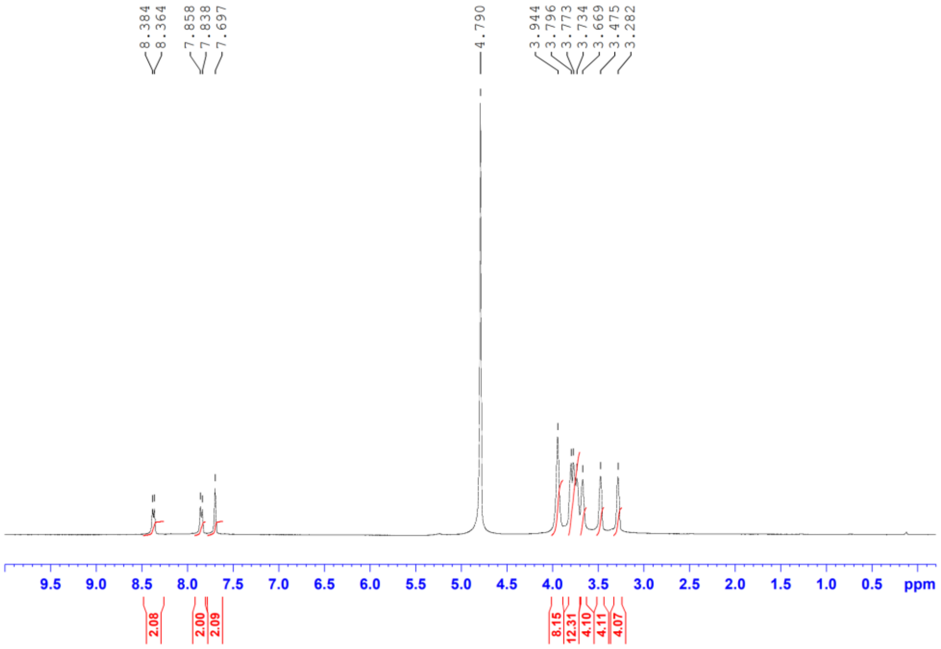

Figure S24. ^{1}H NMR spectrum of **2** in D_2O.

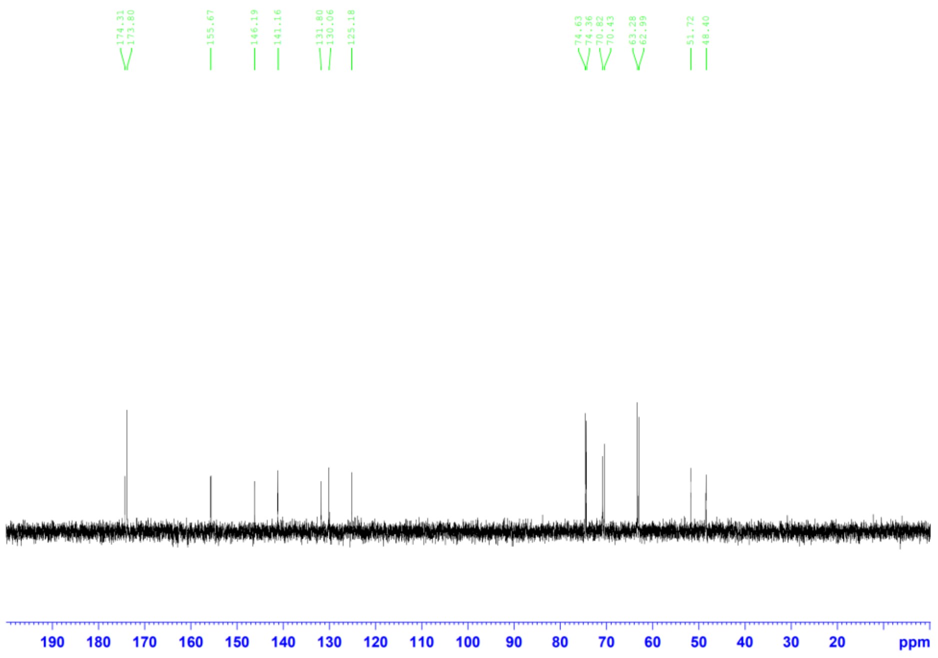

Figure S25. ^{13}C NMR spectrum of **2** in D_2O with $NaHCO_2$ as an internal standard.

6. References

[1] T. J. Liu, K. R. Jansone-Popova, S.; Jiang, D., *xxxx* **2022**, *xxxx*, xxxx.

[2] S. I. Jansone-Popova, A. S.; Bryantsev, V. S.; Sloop, Jr., F. V.; Custelcean, R.; Popovs, I.; Dekarske, M. M.;Moyer, B. A. , *Inorg. Chem.* **2017**, *56*, 5911.

[3] L. J. W. O'Driscoll, D. J.; Bailey, S. W. D.; Visontai, D.; Frampton, H.; Bryce, M. R.; Lambert, C. J. , *Chem. Eur. J.* **2015**, *21*, 3891.

[4] A. Guinier, *Ann. Phys.* **1939**, *11*, 161-237.

[5] aB. N. Ravel, M., *Journal of Synchrotron Radiation* **2005**, *12*, 537-541; bS. I. R. Zabinsky, J. J.; Ankudinov, A.; Albers, R. C.; Eller, M. J., *Phys. Rev. B* **1995**, *52*, 2995-3009.

[6] I. Persson, P. D'Angelo, S. De Panfilis, M. Sandström, L. Eriksson, *Chemistry – A European Journal* **2008**, *14*, 3056-3066.

[7] M. J. Frisch, G. W. Trucks, H. B. Schlegel, G. E. Scuseria, M. A. Robb, J. R. Cheeseman, G. Scalmani, V. Barone, G. A. Petersson, H. Nakatsuji, X. Li, M. Caricato, A. V. Marenich, J. Bloino, B. G. Janesko, R. Gomperts, B. Mennucci, H. P. Hratchian, J. V. Ortiz, A. F. Izmaylov, J. L. Sonnenberg, Williams, F. Ding, F. Lipparini, F. Egidi, J. Goings, B. Peng, A. Petrone, T. Henderson, D. Ranasinghe, V. G. Zakrzewski, J. Gao, N. Rega, G. Zheng, W. Liang, M. Hada, M. Ehara, K. Toyota, R. Fukuda, J. Hasegawa, M. Ishida, T. Nakajima, Y. Honda, O. Kitao, H. Nakai, T. Vreven, K. Throssell, J. A. Montgomery Jr., J. E. Peralta, F. Ogliaro, M. J. Bearpark, J. J. Heyd, E. N. Brothers, K. N. Kudin, V. N. Staroverov, T. A. Keith, R. Kobayashi, J. Normand, K. Raghavachari, A. P. Rendell, J. C. Burant, S. S. Iyengar, J. Tomasi, M. Cossi, J. M. Millam, M. Klene, C. Adamo, R. Cammi, J. W.

Ochterski, R. L. Martin, K. Morokuma, O. Farkas, J. B. Foresman and D. J. Fox, Gaussian 2016.
[8] a) A. D. Becke, *Physical Review A* **1988**, *38*, 3098-3100. b) J. P. Perdew, *Phys. Rev. B* **1986**, *33*, 8822-8824.
[9] M. Kaneko, M. Watanabe, S. Miyashita and S. Nakashima, *RADIOISOTOPES* **2017**, *66*, 289-300.
[10] S. Grimme, J. Antony, S. Ehrlich and H. Krieg, *J. Chem. Phys.* **2010**, *132*, 154104.
[11] M. Dolg, H. Stoll, A. Savin and H. Preuss, *Theoretica chimica acta* **1989**, *75*, 173-194.
[12] D. M. Brigham, A. S. Ivanov, B. A. Moyer, L. H. Delmau, V. S. Bryantsev and R. J. Ellis, *Journal of the American Chemical Society* **2017**, *139*, 17350-17358.
[13] E. Macerata, A. Ossola, W. Panzeri, M. Giola, F. Faroldi, D. A. Tinonin, A. Mele, A. Casnati, M. Mariani, *Solvent Extraction and Ion Exchange* **2018**, *36*, 41-53.
[14] J. Florek, A. Mushtaq, D. Larivière, G. Cantin, F.-G. Fontaine, F. Kleitz, *RSC Advances* **2015**, *5*, 103782-103789.
[15] T. Matsutani, Y. Sasaki, S. Katsuta, *Analytical Sciences* **2021**, *37*, 1603-1609.
[16] Z. Chen, X. Yang, L. Song, X. Wang, Q. Xiao, H. Xu, Q. Feng, S. Ding, *Inorganica Chimica Acta* **2020**, *513*, 119928.
[17] Y. Liu, C. Zhao, Z. Liu, Y. Zhou, C. Jiao, M. Zhang, H. Hou, Y. Gao, H. He, G. Tian, *Journal of Radioanalytical and Nuclear Chemistry* **2020**, *325*, 409-416.
[18] E. A. Mowafy, A. Alshammari, D. Mohamed, *Solvent Extraction and Ion Exchange* **2021**, 1-25.
[19] D. Nomizu, Y. Sasaki, M. Kaneko, M. Matsumiya, S. Katsuta, *Journal of Radioanalytical and Nuclear Chemistry* **2022**, *331*, 1483-1493.
[20] M. Yamaguchi, *Chemical Engineering Research and Design* **1997**, *75*, 447-452.
[21] J. S. Preston, *Hydrometallurgy* **1996**, *42*, 151-167.
[22] C. K. Gupta and N. Krishnamurthy, Extractive Metallurgy of Rare Earths, CRC Press, **2005**.
